# A cost-effectiveness analysis of patiromer in the UK: evaluation of hyperkalaemia treatment and lifelong RAASi maintenance in chronic kidney disease patients with and without heart failure

**DOI:** 10.1186/s12882-023-03088-3

**Published:** 2023-03-09

**Authors:** Thomas Ward, Ruth D. Lewis, Tray Brown, Garth Baxter, Antonio Ramirez de Arellano

**Affiliations:** 1 Health Economics and Outcomes Research Ltd., Rhymney House Unit A Copse Walk Cardiff Gate Business Park, Cardiff, CF23 8RB UK; 2 Health Economics Group, College of Medicine and Health, University of Exeter, Exeter, England; 3 HEOR, CSL Vifor, Staines-Upon-Thames, UK; 4 HEOR, CSL Vifor, Glattbrugg, Switzerland

**Keywords:** Hyperkalaemia, RAASi, Patiromer, Chronic kidney disease, Heart failure, Cost-effectiveness

## Abstract

**Background:**

Chronic kidney disease (CKD) patients with and without heart failure (HF) often present with hyperkalaemia (HK) leading to increased risk of hospitalisations, cardiovascular related events and cardiovascular-related mortality. Renin–angiotensin–aldosterone system inhibitor (RAASi) therapy, the mainstay treatment in CKD management, provides significant cardiovascular and renal protection. Nevertheless, its use in the clinic is often suboptimal and treatment is frequently discontinued due to its association with HK. We evaluated the cost-effectiveness of patiromer, a treatment known to reduce potassium levels and increase cardiorenal protection in patients receiving RAASi, in the UK healthcare setting.

**Methods:**

A Markov cohort model was generated to assess the pharmacoeconomic impact of patiromer treatment in regulating HK in patients with advanced CKD with and without HF. The model was generated to predict the natural history of both CKD and HF and quantify the costs and clinical benefits associated with the use of patiromer for HK management from a healthcare payer’s perspective in the UK.

**Results:**

Economic evaluation of patiromer use compared to standard of care (SoC) resulted in increased discounted life years (8.93 versus 8.67) and increased discounted quality-adjusted life years (QALYs) (6.36 versus 6.16). Furthermore, patiromer use resulted in incremental discounted cost of £2,973 per patient and an incremental cost-effectiveness ratio (ICER) of £14,816 per QALY gained. On average, patients remained on patiromer therapy for 7.7 months, and treatment associated with a decrease in overall clinical event incidence and delayed CKD progression. Compared to SoC, patiromer use resulted in 218 fewer HK events per 1,000 patients, when evaluating potassium levels at the 5.5–6 mmol/l; 165 fewer RAASi discontinuation episodes; and 64 fewer RAASi down-titration episodes. In the UK, patiromer treatment was predicted to have a 94.5% and 100% chance of cost-effectiveness at willingness-to-pay thresholds (WTP) of £20,000/QALY and £30,000/QALY, respectively.

**Conclusion:**

This study highlights the value of both HK normalisation and RAASi maintenance in CKD patients with and without HF. Results support the guidelines which recommend HK treatment, e.g., patiromer, as a strategy to enable the continuation of RAASi therapy and improve clinical outcomes in CKD patients with and without HF.

**Supplementary Information:**

The online version contains supplementary material available at 10.1186/s12882-023-03088-3.

## Background

Hyperkalaemia (HK) is a potentially life-threatening electrolyte abnormality, clinically defined as serum potassium levels above 5.0 mmol/L. Patients with HK are more likely to suffer sudden cardiac arrhythmias, muscle weakness or paralysis [1–4], and are at an increased risk of hospitalisations and mortality [5]. In the clinic, HK is often present in patients with chronic kidney disease (CKD) as a result of renal dysfunction, and is associated with worsening clinical outcomes. Subsequently, CKD patients with HK versus without HK are at increased risk of hospitalisations, cardiovascular-related events and cardiovascular-related mortality [6–14]. Furthermore, HK risk is heightened in patients who are receiving renin–angiotensin–aldosterone system inhibitor (RAASi) treatment, a standard therapy for CKD.

The clinical benefits of using RAASi therapy are well known, with increased cardiovascular and renal protection in cardiorenal patients. In CKD, RAASi use has been shown to decrease blood pressure and proteinuria [15], reduce the risk of kidney failure, cardiovascular morbidity and cardiovascular-related and all-cause mortality [16], and slow CKD progression [17]. Despite RAASi having a significant impact on slowing CKD progression and reducing cardiovascular events, its use in the clinic is often suboptimal and treatment is frequently discontinued due to its association with HK [18, 19], resulting in worsening clinical outcomes in both CKD and heart failure (HF) populations [8, 11, 20–26]. In the UK, major adverse cardiac events (MACE) and mortality were consistently higher in patients receiving sub-optimal RAASi dose (< 50% of the recommended RAASi dose) [19]. Subsequently, these patients are at significant risk of hospitalisation, significantly impacting resource use and overall health care costs [27, 28].

Patiromer, a non-absorbed cation exchange polymer, has demonstrated effectiveness in cardiorenal patients receiving RAASi therapy, both in terms of reducing potassium levels and enabling the initiation and up-titration of RAASi in patients at risk of HK [29–32]. The objective of this study is to evaluate the cost-effectiveness of patiromer in the UK healthcare setting. A further objective is to evaluate the relationship between HK incidence and optimal RAASi management, and lifetime economic outcomes.

## Method

### Patiromer OPAL-HK trial

The modelling approach has previously been published [33] and was developed in order to extrapolate results from the OPAL-HK trial. This trial was used to assess the efficacy and safety of patiromer and was an international, multicentre, single blind, phase III clinical trial investigating the acute treatment of HK, and the ongoing maintenance of normokalaemia. The study was carried out in two sequential parts over 12 weeks.

The treatment phase (Part A) was a single blind, single arm trial of patiromer for four weeks. Patients were eligible for inclusion if they had stage 3 or 4 CKD, a serum potassium level of 5.1 to < 6.5 mmol/L and were receiving a stable RAASi dose. At the time of screening, patients were assigned to receive a starting dose of 4.2 g twice daily or 8.4 g twice daily depending on the severity of HK. In this phase, RAASi doses were not adjusted; they were only discontinued if the potassium level was ≥ 6.5 mmol/L (≥ 5.1 mmol/L if on the maximum permitted patiromer dose).

The withdrawal phase (Part B) was a placebo controlled, single blind, randomised withdrawal trial of patiromer for eight weeks. The objective of the withdrawal phase was to evaluate the effect of withdrawing patiromer on serum potassium control and to assess whether chronic treatment with patiromer prevents the recurrence of HK.

### Cost-effectiveness model

A Markov cohort model was developed to assess the health economic impact of patiromer therapy in comparison to standard of care (SoC) in controlling HK in advanced CKD patients with and without HF. The model was designed to predict the natural history of CKD and HF and quantify the costs and benefits associated with the use of patiromer for serum potassium management from a payer perspective in the UK. CKD and HF are chronic and progressive diseases associated with increased risk of mortality. As such, a lifetime horizon was modelled in line with technology assessment guidelines [34, 35]. A monthly cycle length was adopted and disease progression followed over a lifetime.

### Model structure and disease progression

**Fig. 1:**
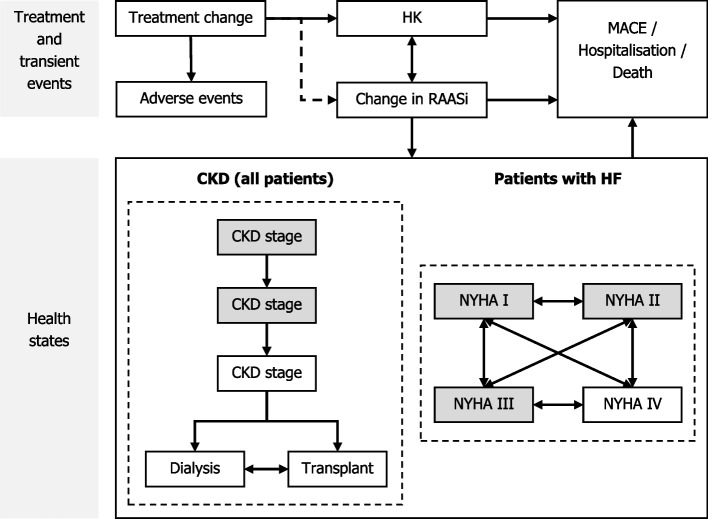
Model flow diagram. States highlighted in grey represent starting health states

Patients enter the model (Fig. 1) with either CKD alone or CKD with HF. The progression of CKD patients was modelled via transitions to more progressed CKD stages and eventually end-stage renal disease (ESRD), comprising of separate dialysis and transplant states. Similarly, the progression of HF in CKD + HF patients was modelled via transitions between New York Heart Association (NYHA classifications (I to IV) [36–39]. Both CKD and HF are modelled independently, with progression through health states in one not impacting progression through health states in the other, except for those exiting the model in the death health state. As a simplifying assumption, patients without HF at model initiation do not develop HF during the modelled time horizon. The starting distribution of patients is presented in Table 1, alongside baseline age and sex, whilst baseline rates of CKD and HF disease progression are described further in Supplemental Appendix A.

**Table 1:** Starting health state distribution and baseline patient characteristics

	**Mean**	**SE**	**Source**
**Starting health state distribution**			
Proportion with HF	41.98%	-	OPAL-HK CSR [43]
Proportion CKD stage 3	55.14%	3.19%	OPAL-HK CSR; CKD stage 2 patients included [43]
Proportion CKD stage 4^a^	44.86%	3.19%	OPAL-HK CSR [43]
Proportion CKD stage 5 ^a^	0.00%	0.00%	
Proportion NYHA I	18.63%	3.85%	
Proportion NYHA II	64.71%	4.73%	
Proportion NYHA III	16.67%	3.69%	
Proportion NYHA IV	0.00%	0.00%	
Proportion normokalaemia (K + ≤ 5)	0.00%	0.00%	Assumed
Proportion HK (K + > 5 to ≤ 5.5)	0.00%	0.00%	
Proportion HK (K + > 5.5 to ≤ 6)	81.35%	3.17%	OPAL-HK CSR; distributed across upper threshold categories in line with published data [43]
Proportion HK (K + > 6)	18.65%	3.17%	
**Patient characteristics**			
Age (years)	65.30	0.89	OPAL-HK CSR [43]
Proportion female	0.46	0.05	

*CKD* Chronic kidney disease, *HF* Heart failure, *K* + Potassium, *NYHA* New York Heart Association

^a^Note in the OPAL-HK CSR, patients were described only as “stage 4 or worse” [43]. The proportion of patients pre-RRT in stage 5 is thus unknown and here taken as 0

As the simulated cohort progresses through the model, the value of alternative treatments is captured through the occurrence of HK events, changes in RAASi use and treatment discontinuation. The likelihood of other events (MACE, hospitalisation and mortality) is also predicted and is impacted directly by a patient’s health state (i.e., CKD and HF) and by RAASi use and HK incidence (i.e., potassium level); baseline rates may be found in Supplemental Appendix A [23, 40–42]. MACE was defined as events of coronary heart disease, HF, ischemic stroke, and peripheral arterial disease leading to hospitalisation. Hospitalisation was defined as any hospitalisation. The probability of MACE, hospitalisation and mortality, stratified by disease severity, are estimated for a CKD-only and HF-only patient, and the higher of the two probabilities are then applied for the cohort with CKD + HF. In both cohorts, where all-cause mortality estimates from UK-specific life tables exceeded mortality estimates based on comorbidities and RAASi use, the greater mortality rate was assumed. As a simplifying assumption based on results of the OPAL-HK trial, there is assumed to be no significant difference in the likelihood of therapy-attributable adverse events between treatment and comparator arms, and they are therefore not incorporated into the model.

### Hyperkalaemia

**Fig. 2:**
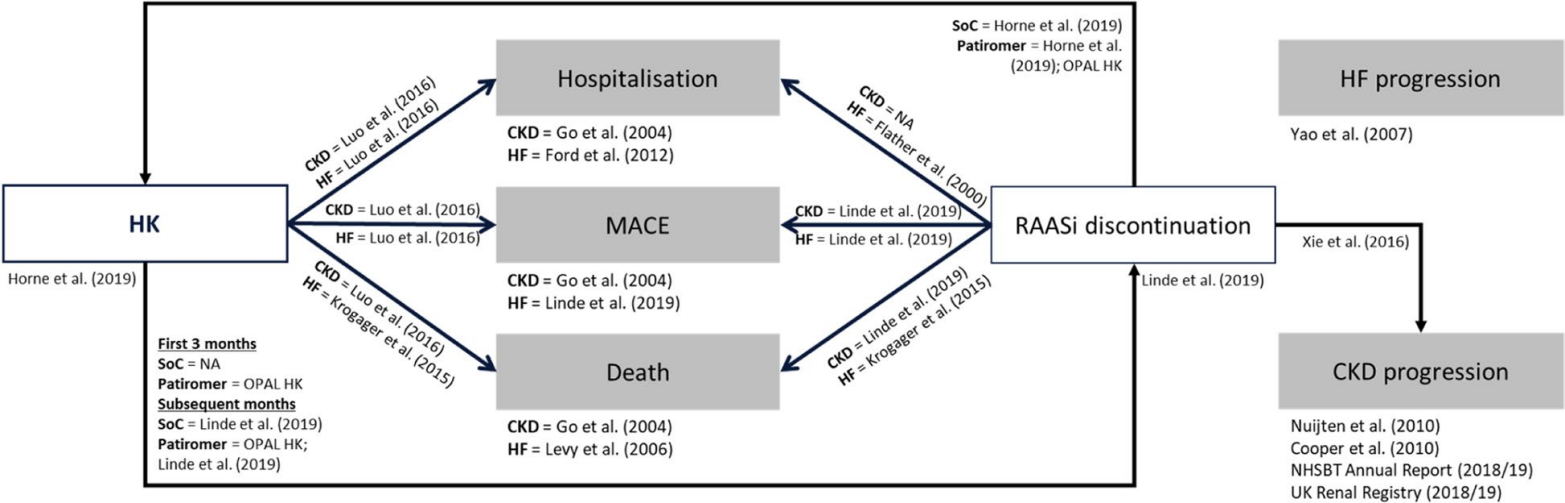
Influence of RAASi use and HK events on disease progression and events. References below each box describe the baseline probabilities/rates; references alongside arrows describe the influence of one disease component on the other, with influences applied to the baseline probabilities rates

The occurrence of HK was categorized as a serum potassium level greater than 5 mmol/l, consistent with the definitions used in the OPAL-HK trial and widely accepted in the broader HK literature [29, 44]. Events were further stratified by severity (i.e., 5–5.5 mmol/l, 5.5–6 mmol/l and > 6 mmol/l). During the first three months of the modelled time horizon, incident HK events are predicted based on data from the OPAL-HK trial [29, 45]. For all subsequent months, annual rates of HK were obtained from Horne et al. (2019) and applied to the SoC arm [46]. Hazard ratios relating to reduced (or increased) incidence in those receiving patiromer in subsequent years were obtained from the OPAL-HK trial and applied to the annual rates of HK obtained from Horne et al. (2019). HK event rates are summarised in Table 2. Increased potassium levels negatively impact the incidence of MACE, hospitalisation and death (Fig. 2); the magnitude of these impacts is further described in Supplemental Appendix A.

**Table 2:** HK incidence

Time applied	Potassium level	Monthly probability				Source
		**Patiromer**		**SoC**		
		**Mean**	**SE**	**Mean**	**SE**	
Month 1	K + > 5 to ≤ 5.5	21.13%	3.32%	21.13%	3.32%	OPAL-HK CSR; distributed across threshold categories in line with published data [43, 46]
	K + > 5.5 to ≤ 6	1.66%	1.04%	1.66%	1.04%	
	K + > 6	0.38%	0.50%	0.38%	0.50%	
Month 2 & 3	K + > 5 to ≤ 5.5	14.00%	4.68%	15.00%	4.81%	OPAL-HK CSR [43]
	K + > 5.5 to ≤ 6	6.10%	3.23%	25.22%	5.86%	
	K + > 6	1.40%	1.58%	5.78%	3.15%	
Subsequent months^a^	K + > 5 to ≤ 5.5	0.543%	0.054%	1.158%	0.116%	Horne et al. (2019); 'OPAL-HK CSR [43, 46]
	K + > 5.5 to ≤ 6	0.022%	0.002%	0.092%	0.009%	
	K + > 6	0.005%	0.001%	0.021%	0.002%	

*HK* Hyperkalaemia, *RAASi* Renin–angiotensin–aldosterone system inhibitor, *SE* Standard error, SoC Standard of care

^a^SoC probabilities informed by HK recurrence rates observed in Horne et al. (2019) with recurrence events distributed in line with the distribution of initial HK events across potassium categories; patiromer estimates informed by Horne et al. (2019) after application of a HR based on OPAL-HK data from months 2 and 3; SE assumed as 10% of mean

### RAASi use

In both treatment arms, all patients are initiated in the model on RAASi and are assumed to be receiving a maximum dose. Down-titration to a sub-maximal dose, or discontinuation of RAASi treatment (from any dose) may occur. RAASi use favourably impacts the progression of CKD and the incidence of MACE, hospitalisation and death (Fig. 2), with an increase in the incidence of HK; the magnitude of these impacts is further described in Supplemental Appendix A [23, 36–42, 46–50].

The proportion of patients still on RAASi at the end of the first month is specified for both arms and based on OPAL-HK trial data. For the patiromer arm, this proportion relates only to those that have achieved response, with the remaining patients assumed to be receiving RAASi therapy in line with the SoC arm. Rates of RAASi discontinuation and down-titration are taken from the OPAL-HK trial for months 2 and 3 [43]. From month 4 onwards, potassium level dependent RAASi discontinuation and down-titration rates were taken from Linde et al. (2019) and applied to the SoC arm [23]. Hazard ratios relating to reduced (or increased) rates of discontinuation/down-titration in those receiving patiromer in subsequent months were obtained from the OPAL-HK trial and applied to the rates from Linde et al. (2019). To reflect the impermanent nature of RAASi treatment changes in clinical practice, patients could return to optimal RAASi use independent of their potassium level with a monthly probability of 3.51% [23]. Due to a lack of relevant data, patients who down-titrated RAASi use were assumed to not return to maximum use. RAASi discontinuation and down-titration rates are summarised in Table 3.

**Table 3:** RAASi discontinuation, down-titration and up-titration, by potassium category

	**Monthly probability of RAASi max discontinuation (%)**		**Monthly probability of RAASi max down-titration (%)**		**Monthly probability of RAASi sub-max discontinuation (%)**		**Source**
	**SoC**	**Patiromer**	**SoC**	**Patiromer**	**SoC**	**Patiromer**	
**Month 2–3**	34.438% (6.589%)	3.336% (2.421%)	35.549% (6.589%)	0.000% (0.000%)	34.438% (6.589%)	3.336% (2.421%)	OPAL-HK [43]
**Subsequent months**							
**K + ≤ 5**	2.600% (0.009%)	0.181%	1.800% (0.026%)	1.800%	2.600% (0.009%)	0.181%	Linde et al. (2019) [23]
**K + > 5 to ≤ 5.5**	3.029% (0.102%)	0.211%	2.617% (0.102%)	2.617%	3.029% (0.102%)	0.211%	
**K + > 5.5 to ≤ 6**	4.547% (0.230%)	0.319%	5.306% (0.230%)	5.306%	4.547% (0.230%)	0.319%	
**K + > 6**	10.000% (0.663%)	0.721%	8.900% (0.638%)	8.900%	10.000% (0.663%)	0.721%	

*RAASi* Renin–angiotensin–aldosterone system inhibitor, *K* + Potassium, *SE* Standard error, *SoC* Standard of care

Note: Complete derivation described further in Supplemental Appendix A

### Treatment

The model evaluates patiromer use against current SoC, as previously published. [33] It should be noted that modelling SoC is particularly challenging, due to the considerable heterogeneity associated with HK pathogenesis, methods to correct and manage potassium levels (particularly non-pharmacological interventions, and variable levels of adherence to pharmacological methods), and patient responses to such interventions. As such, SoC has been defined consistently with the broad definitions used in the OPAL-HK study, where SoC can be considered acute management for the correction of potassium and lifestyle interventions for the background maintenance of potassium (e.g., dietary intervention and modification of concomitant medications).

All patients initiated in the treatment arm were assumed to receive patiromer for at least one month. At the end of the first month, patients were stratified into those that do (60.93%) and do not (39.07%) respond to treatment. Within the patiromer arm, those that respond to treatment continue to receive patiromer and the associated event risks. Those that do not respond to patiromer cease treatment and incur the risk of events in line with SoC (i.e., assuming no legacy effect of patiromer treatment). For the SoC arm, treatment with SoC could not be discontinued. Beyond month 1, patients receiving patiromer could discontinue at a constant monthly rate of 10.33% based on the OPAL-HK trial, or if they reached ESRD; subsequently incurring event risk in line with the SoC arm. Patients repeated treatment if their potassium levels were equal to or exceeded 5.5–6 mmol/l in subsequent months after discontinuation.

### Costs and utilities

Supplemental appendix B summarises the direct medical costs (2019–20 GBP) applied to modelled health states and events. UK-specific cost data were used, and all costs were inflated to 2019/20 values [51–68]. Supplemental appendix C summarises the utilities (and disutilities) applied to modelled health states (and events) [54, 55, 69–72]. Utility estimates were broadly informed by a recent National Institute for Health and Care Excellence (NICE) technology appraisal [61]. All cost and utility outcomes were discounted at an annual rate of 3.5% in line with UK health technology assessment guidelines.

### Analysis

#### Base cost-effectiveness analysis

The model was used to evaluate the lifetime impact of patiromer use against SoC for the treatment of HK in patients with CKD with and without HF, as previously published. [33] Modelled outcomes focused on health care costs, life years and quality-adjusted life years (QALYs), with comparisons between treatments made using the incremental cost-effectiveness ratio (ICER).

Probabilistic sensitivity analysis was undertaken to evaluate uncertainty in clinical and economic outcomes. Patient characteristics and demographics were sampled using a normal distribution, probabilities and utility and disutility values were sampled using a beta distribution, and costs, hazard ratios and odds ratios were sampled using a gamma distribution. Deterministic sensitivity analysis was also undertaken to assess the impact of individual model parameters on model outcomes; the most influential and uncertain input parameters were incorporated in the analysis.

#### Impact of HK incidence

The incidence of HK can vary significantly across individual patients and so, to evaluate the potential impact of HK on total cost, QALY and life year outcomes, the annual rates of HK were varied over a meaningful range (0–0.5) and outcomes compared over a patient’s lifetime. The model stratifies HK events by severity and so, to incorporate an evaluation of the impact of HK severity, event rates for potassium levels 5–5.5 mmol/l, 5.5–6 mmol/l and > 6 mmol/l were evaluated separately. This scenario is evaluated without the impact of patiromer treatment, assuming input values in line with the SoC arm. All other model parameters remained as in the base cost-effectiveness analyses, and results are presented as incremental results versus an assumed scenario of no HK incidence for the evaluated potassium level.

#### Value of optimal RAASi control

Management of HK often involves the discontinuation or down-titration of RAASi therapy. The enablement of RAASi therapy is extremely important for the clinical management of patients with CKD with or without HF. To illustrate the potential lifetime benefits associated with optimal RAASi control, we evaluate two hypothetical patient cohorts, one that maintains optimal RAASi control over their entire lifetime (from the point of model initiation), and one that is not ever managed with RAASi therapy (or at least, not managed with RAASi therapy from the point of model initiation). We evaluate each of these management approaches in patient cohorts aged 40, 50, 60 and 70, utilising different starting CKD health states (CKD stages 3, 4 and 5) and assuming patients do or do not suffer from HF.

Given a strong association between age and ESRD treatment modalities (dialysis and transplant) and their outcomes, the likelihood of transplant and the likelihood of death from ESRD are modified for each age cohort; input parameters are detailed in Table 4. This scenario is evaluated without the impact of patiromer treatment, assuming input values in line with the SoC arm. All other model parameters remained as in the base cost-effectiveness analyses.

**Table 4:** Age-dependent ESRD input parameters

Parameter	Age				Source
	**40**	**50**	**60**	**70**	
Monthly probability of transplant from CKD stage 5	2.15%	1.68%	0.18%	0.18%	NHSBT [38]; Renal Registry [73]
Monthly probability of transplant from dialysis	0.70%	0.55%	0.06%	0.06%	NHSBT [38]; Renal Registry [73, 74]
Monthly probability of death from dialysis	0.18%	0.37%	0.61%	1.23%	Renal Registry [74]
Monthly probability of death from transplant	0.07%	0.18%	0.32%	0.55%	NHSBT [38]; Karim et al. (2014) [75]

*CKD* Chronic kidney disease, *NHSBT* National Health Service Blood and Transplant

Note: Examples of the derivation of the above inputs are provided in Supplemental Appendix A

## Results

### Base cost-effectiveness analysis

Base case cost-effectiveness results are presented in Table 5. Treatment with patiromer was associated with an increase in discounted life years (8.93 versus 8.67) and an increase in discounted QALYs (6.36 versus 6.16). Incremental discounted costs were predicted at £2,973 per patient, with an incremental cost-effectiveness ratio of £14,816 per QALY gained. Discounted incremental costs were predominantly driven by an initial increase in costs associated with patiromer treatment, increased costs of disease management due to extension of life and reductions in RAASi titration costs over the patient’s lifetime, as a consequence of improved RAASi enablement.

**Table 5:** Cost-effectiveness results

	**Patiromer**	**SoC**	**Incremental**
**Discounted results**			
Total costs (£)	£116,675	£113,701	£2973
Treatment	£1283	£0	£1283
HK	£1091	£1287	-£196
CKD	£27,535	£26,628	£907
RRT	£56,877	£56,155	£721
MACE	£9227	£9280	-£53
Hospitalisation	£18,684	£18,226	£458
RAASi drug usage	£153	£130	£23
RAASi titration	£1824	£1995	-£170
Total life years	8.935	8.670	0.264
Total QALYs	6.356	6.156	0.201
ICER (£/QALY)	-	-	£14,816
**Undiscounted results**			
Total costs	£168,834	£164,306	£4528
Total life years	11.685	11.321	0.364
Total QALYs	8.176	7.904	0.272
ICER (£/QALY)	-	-	£16,672

*CKD* Chronic kidney disease, *HK* Hyperkalaemia, *ICER* Incremental cost-effectiveness ratio, *QALY* Quality-adjusted life year, *RAASi* Renin–angiotensin–aldosterone system inhibitor, *RRT* Renal replacement therapy, *SoC* Standard of care

Patients remained on patiromer treatment for an average of 7.7 months, with treatment associated with a reduction in the rate of adverse clinical event incidence and a delay in CKD disease progression. However, due to patients in the patiromer arm observing an increased life expectancy, the total incidence of hospitalisation, dialysis and kidney transplantation was greater, despite rates being reduced. Per 1,000 patients, patiromer compared to SoC was associated with 218 and 50 fewer HK events, when evaluating potassium levels at the 5.5–6 mmol/l and > 6 mmol/l levels, respectively. Patiromer when compared with SoC was also associated with 165 fewer RAASi discontinuation episodes and 64 fewer RAASi down-titration episodes. Subsequently, improvements in RAASi management enabled an overall increase in the time it took patients to reach renal replacement therapy (RRT), resulting in a similar number of incident dialysis and transplant episodes, despite improvements in life extension which inherently increase the likelihood of such incidence.

**Fig. 3:**
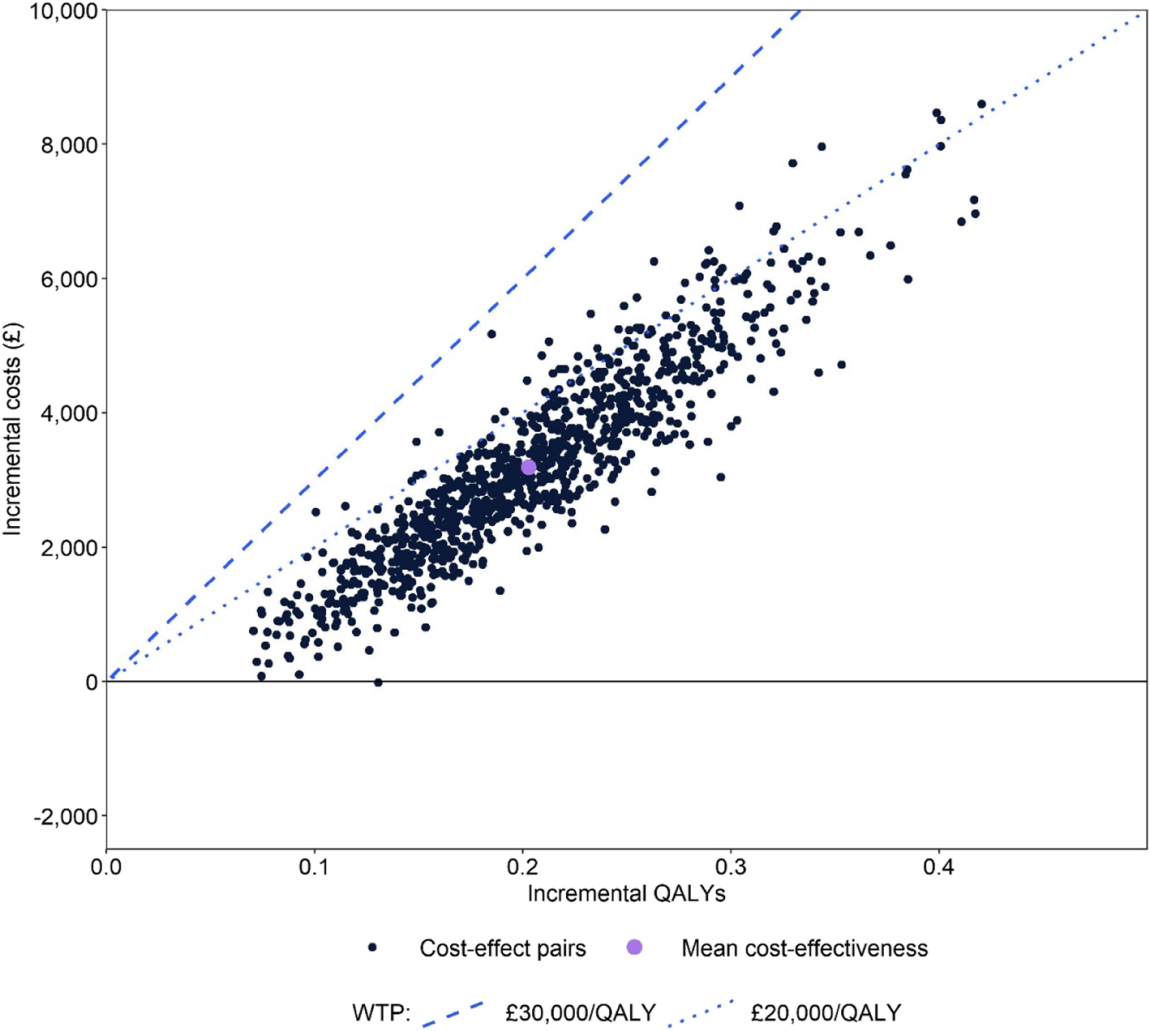
Probabilistic sensitivity analysis

Probabilistic sensitivity analysis is presented in Fig. 3 and supports the conclusions of the base case analysis. Treatment with patiromer was estimated to have a 94.5% and 100% chance of cost-effectiveness compared to SoC when evaluated at willingness-to-pay thresholds of £20,000/QALY and £30,000/QALY in the UK. One-way sensitivity analyses, presented in Supplemental Appendix D demonstrates that cost-effectiveness conclusions are relatively robust to changes in individual parameters, with results most sensitive to rates of discounting, the modelled time horizon, baseline patient age, the magnitude of the impact of RAASi use on CKD progression, and RAASi and treatment discontinuation.

### Impact of HK incidence

**Fig. 4:**
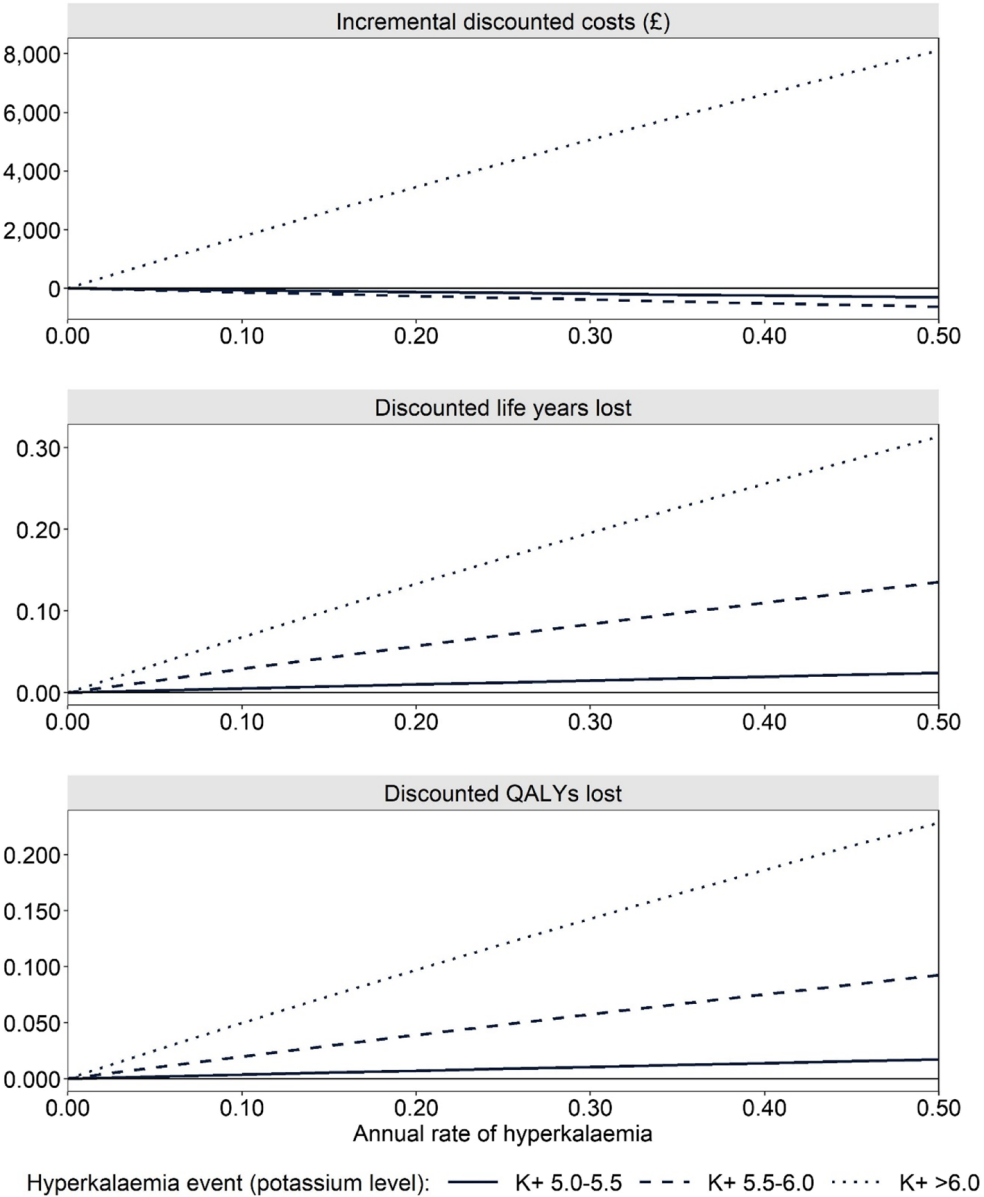
Impact of changes in the annual rate of HK on costs, QALYs and life years (compared to no HK incidence). All other inputs remain as in the base case cost-effectiveness analysis

The impact of HK incidence is presented in Fig. 4. Increasing HK incidence was associated with QALY and life year reductions, with increases in the most severe HK events resulting in the greatest losses. Increasing the annual rate of HK to 0.5 resulted in QALY losses of 0.017, 0.093 and 0.229 per patient, when compared to a similar cohort in which no HK incidence was observed, for potassium levels 5–5.5 mmol/l, 5.5–6 mmol/l and > 6 mmol/l, respectively. Life year and QALY reductions come as a consequence of HK being associated with additional morbidity and mortality. With regards to costs, there are three core components associated with HK incidence that influence total cost accrual: the cost of managing the individual HK event (£0, £223.11 and £2,933.49 for potassium levels 5–5.5 mmol/l, 5.5–6 mmol/l and > 6 mmol/l, respectively), increased morbidity associated with HK (increasing costs) and, increased mortality associated with HK (reducing costs). Increasing the rate of the most severe HK events (i.e., potassium > 6.0 mmol/l) resulted in increased lifetime per-patient costs of up to £8,109 when event rates were increased to 0.5 per year (predominantly due to the increased cost associated with HK management). In contrast, increasing the rate of less severe HK events (i.e., potassium levels ≤ 6.0 mmol/l) resulted in reduced lifetime per-patient costs (albeit marginal cost reductions). Cost reductions were attributed to the much lower cost of managing these HK events (compared to severe HK events) and the reduction in life expectancy, resulting in less time for patients to accrue costs of general disease management associated with CKD and HF.

### Value of optimal RAASi control

**Fig. 5:**
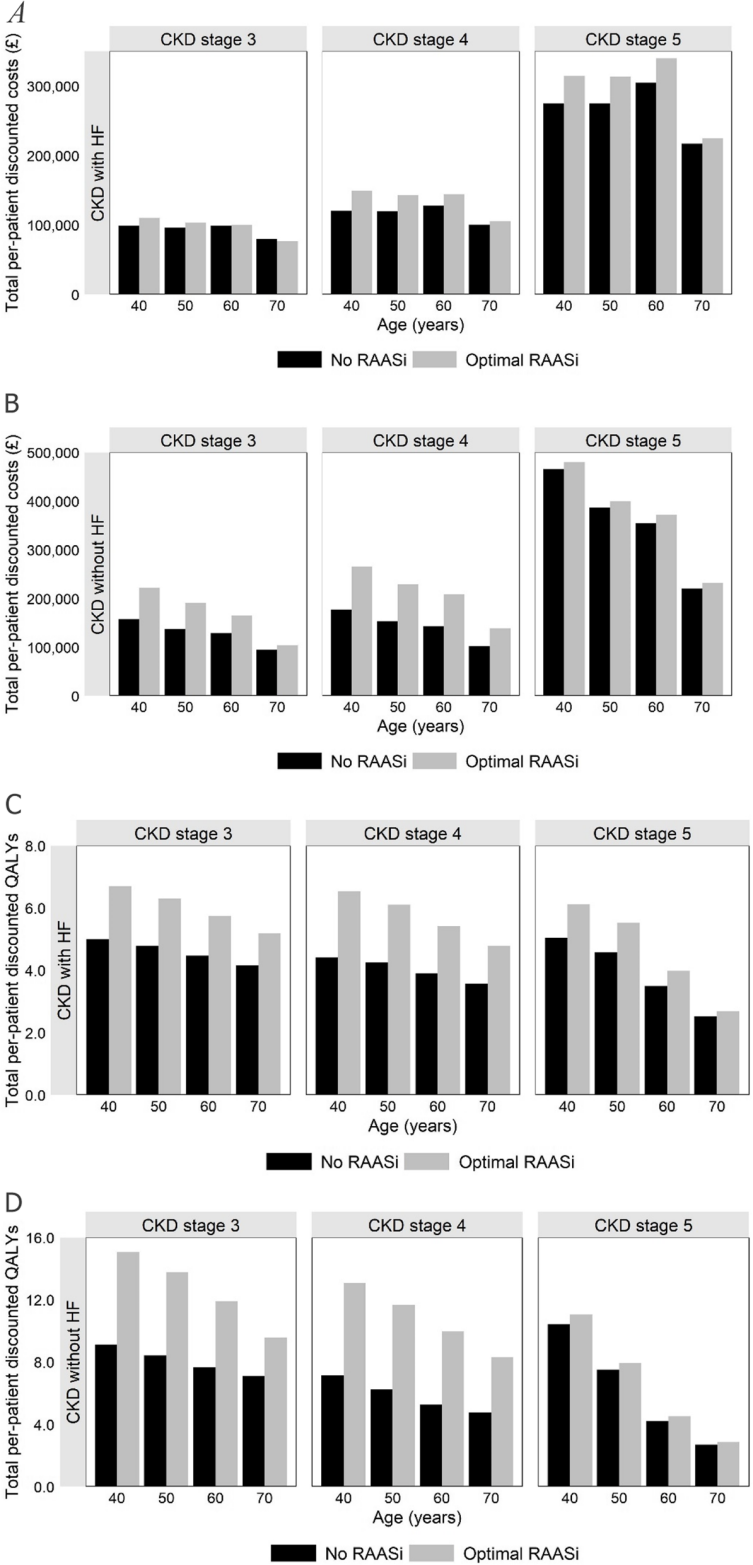
The impact of lifetime optimal RAASi management (compared to no RAASi use) and the association of outcomes with patient’s baseline age, starting CKD stage and HF disease status. **A**: Total per-patient discounted costs in patients with CKD and HF; **B**: Total per-patient discounted costs in patients with CKD without HF; **C**: Total per-patient discounted QALYs in patients with CKD and HF; **D**: Total per-patient discounted QALYs in patients with CKD without HF; Note: All other inputs remain as in the base case cost-effectiveness analysis

The value of maintaining optimal RAASi control is presented in Fig. 5, in terms of total costs and QALYs, with results presented for patients with optimal RAASi use and patients with no RAASi use. Patients with optimal RAASi management were consistently estimated to observe greater quality-adjusted life expectancy, with the largest differences between optimal RAASi management and no RAASi management typically observed in patients without HF, those of younger age and those starting in less severe CKD stages. These groups typically gain the most due to their greater propensity to avoid ESRD and its consequences. As expected, those with both CKD and HF observe much lower QALY gains than those with CKD alone, where HF-related mortality is a dominant factor and there is less time available for RAASi use to influence outcomes.

In those without HF, optimal RAASi management is typically associated with greater cost due to extension of life and the increased amount of time managing CKD and ESRD. Since, in cases where a technology increases survival in people for whom the NHS is currently providing care that is expensive, NICE may consider, alongside the reference-case analysis, a non-reference-case analysis with the background care costs removed. [76] Hence, the greater costs associated with RAASi management may be exempt during a NICE technology appraisal process.

A similar relationship is observed amongst those with HF, although the differences are less pronounced (and in the case of some CKD stage 3 patients, reversed), due to smaller gains in life expectancy, and the increased costs associated with disease management being partially offset by the avoidance of MACE and hospitalisation events.

Across non-HF and HF populations, optimal RAASi management in younger patients is typically associated with greater cost due to increased life expectancy, and subsequently, a greater amount of time spent managing CKD. An exception to this is observed in patients with HF starting in CKD stage 5, where the non-linear relationship between ESRD modalities (e.g., transplant eligibility), ESRD transition rates and death play a more influential role given patients immediate proximity to these health states. Total costs are on average greater in those without HF, than those with HF, for similar reasons. In contrast, total costs increase as the starting CKD stage worsens due to a closer proximity of patients to resource intensive ESRD health states.

Importantly, these results highlight the complexity of the economic relationships observed when modelling HK in a cohort of patients with CKD with or without HF. Inherently, these are complicated conditions with treatment and outcomes from one disease component influencing treatment and outcomes in another, and vice versa, often leading to results which require additional interpretation before appearing intuitive.

## Discussion

This study evaluates the cost-effectiveness of patiromer for the treatment of HK in patients with CKD with or without HF and demonstrates that patiromer is a cost-effective treatment in the UK setting. Further, this study adds to the published literature by undertaking extensive sensitivity analyses exploring the impact of HK and RAASi use on UK patient lifetime outcomes. To our knowledge, this is the first study to estimate the lifetime economic impact of optimal RAASi use in HK patients with CKD with or without HF.

The analysis in this study demonstrates that the avoidance of HK and the maintenance of optimal RAASi therapy is associated with both life year and QALY gains, and in some scenarios cost-savings. These findings are in accordance with other studies evaluating the benefits of HK management; HK avoidance and RAASi enablement, in CKD and HF. [77, 78] Evans M et al. modelled the natural history of CKD in order to demonstrate the relationship between potassium levels, RAASi therapy and long-term clinical outcomes in CKD patients. [77] Authors demonstrated that normalisation of potassium levels and optimal RAASi use was associated with delayed CKD progression and RRT initiation, better quality of life, increased survival and cost savings. In another study, the health and economic benefits of HK normalisation and continuation of RAASi therapy was evaluated in HF patients. [78] Analysis showed that patients who maintained normal potassium levels and RAASi use had increased life expectancy, QALYs, cost savings and associated net monetary benefit over a lifetime horizon. Together, these results highlight the importance of implementing a successful strategy for HK management and maintenance of RAASi therapy and should be actively pursued given that both HK treatment and in particular RAASi use are relatively inexpensive in the UK.

Patiromer, a non-absorbed polymer which binds to potassium in exchange for calcium within the gastrointestinal tract, has been demonstrated to be cost effective in the UK as a treatment option for HK patients. [79] Clinical trials have demonstrated the benefits of patiromer as an effective, well tolerated and fast acting strategy to normalise potassium levels, enable RAASi therapy and allow long-term management in patients with HK. [29, 30, 80] Moreover, ongoing studies of patiromer are underway to determine patient reported outcomes as a measure of quality of life and mortality in the RELIEHF clinical trial. [81] Findings from such trials will further inform cost-effectiveness modelling and our understanding of the effect of patiromer treatment, HK incidence and RAASi therapy on increased survival and its impact on the quality of life of patients living with chronic diseases. Furthermore, patiromer has been recommended in the UK for the treatment of HK in patients with CKD or HF. [79] Nevertheless, in the clinic HK is often managed by down-titration or discontinuation of RAASi therapy, resulting in worsening clinical outcomes [22, 47, 82] and increased burden on the healthcare systems, with increased hospitalisations and resource use.

Current economic evaluations often do not take into consideration indirect health care consequences, such as the benefits associated with reduced hospitalisations, and instead, assume that capacity is not an issue. Despite the UK adopting a national “healthcare for all” health service approach, significant increases in need over recent years have resulted in a healthcare service stretched beyond its capacity. As such, the benefits of interventions which keep patients out of hospital are likely underestimated. For instance, reducing hospitalisations would free up resource use which could impact on the cost-effectiveness of other interventions. Furthermore, the additional resource available would allow capacity for other health care to be provided.

This is particularly relevant given the current challenges healthcare systems are facing, during the Covid 19 pandemic. In the UK, NHS hospitals were already operating at 90% capacity pre-pandemic. [83] Requirement for in-patient care has significantly increased over the last two years and adjusting to free-up the number of hospital beds to meet demand is challenging. [84] In England, one of the approaches taken was cancellations of elective surgery at the detriment of non-Covid-19 patients’ health, resulting in an increased length of waiting lists for patients needing healthcare. [85–89] Subsequently, the current challenge for healthcare managers is to obtain sufficient hospital capacity to care for COVID-19 patients whilst also being able to continue treatment for non-COVID 19 patients. Our results suggest an alternative approach to increasing hospital bed capacity, through improved HK management. In our model, normalisation of potassium levels and continuation of RAASi therapy resulted in reductions in the rate of all adverse clinical outcomes and time spent in the healthcare system due to RAASi management issues.

The results of this study also highlight the complexity of the modelled relationships, which attempt to capture outcomes associated with several multi-faceted disease areas. Not only are these complex disease areas, but each has the potential to impact the other through the influence of either treatment or outcomes. Only by further exploring the impact of HK incidence and lifetime RAASi use on model outcomes and providing this additional interpretation, do these relationships and interactions become more apparent and intuitive. Models are inherently designed to explore such uncertainty, however, without confirmation of their ability to model these dynamic relationships, through either validation to large observational studies or validation with clinical experts, there will remain doubt over modelled results. As such, future research may focus on first extending model validation beyond the core model application (for instance cost-effectiveness of a specific treatment in a specific static setting) to further fully validate model relationships and scenarios which might only be realised when undertaking exploratory analyses, and second, to provide a more comprehensive set of guidelines for model validation processes which direct the validation of complicated disease areas beyond the ‘base case’ setting.

Limitations of this study are mainly due to the relative paucity of the literature. In the base case cost-effectiveness analysis, extrapolation of outcomes was based on a 3-month trial, which is inherently uncertain. Furthermore, whilst the influence of RAASi management on CKD and HF outcomes is well accepted in the published literature, the magnitude of such influence is more uncertain. In addition, our exploration of optimal RAASi use scenarios only captures the influence of age on some dialysis and transplant input parameters, due to limitations in available data. It is likely that modification of other clinical parameters, particularly in relation to the influence of age and disease status would more accurately reflect real-world clinical practice. However, this study can be seen as an indicative first step in quantifying the value of optimal RAASi use.

## Conclusions

In summary, findings from this study highlight the value of both HK normalisation and RAASi maintenance in CKD patients with and without HF. HK treatment was associated with a reduction in overall clinical event incidence and a delay in CKD disease progression. In addition, the value of lifetime optimal RAASi control was associated with increased QALY and life year gains, and in some scenarios cost savings. Together, these results support the guidelines which recommend HK treatment, e.g., patiromer, as a strategy to enable the continuation of RAASi therapy and improve clinical outcomes in CKD patients with and without HF.

## Supplementary Information

**Additional file 1: ** This appendix provides details of disease progression data utilised in the model.

**Additional file 2: ** This appendix provides details of cost data utilised in the model.

**Additional file 3: ** This appendix provides details of utility and disutility input parameters utilised in the model.

**Additional file 4: ** This appendix provides details of additional results not presented in the main manuscript body.

## Data Availability

All data generated or analysed during this study are included in this published article and the supplementary material.

## Abbreviations

CKD: Chronic kidney disease
ESRD: End-stage renal disease
HF: Heart failure
HK: Hyperkalaemia
ICER: Incremental cost-effectiveness ratio
K +: Potassium
MACE: Major adverse cardiac event
NHSBT: National Health Service Blood and Transplant
NYHA: New York Heart Association
QALY: Quality-adjusted life year
RAASi: Renin–angiotensin–aldosterone system inhibitor
RRT: Renal replacement therapy
SE: Standard error
SoC: Standard of care
