# Supplementary material for: A cost-effectiveness analysis of patiromer in the UK: evaluation of hyperkalaemia treatment and lifelong RAASi maintenance in chronic kidney disease patients with and without heart failure
Source: BMC Nephrol. 2023 Mar 9;24:47. doi: 10.1186/s12882-023-03088-3 (PMC9995261; doi:10.1186/s12882-023-03088-3)
Supplement: Supplementary file 1 — Additional file 1: This appendix provides details of disease progression data utilised in the model. [file 12882_2023_3088_MOESM1_ESM.docx]

**Supplemental Appendix A**

This appendix provides details of disease progression data utilised in the model.

## **Baseline patient characteristics**

Additional patient characteristics beyond those specified in the main manuscript document are required to estimate the likelihood of death in patients with HF. Table 1 summarises these additional patient characteristics. All patient characteristics described in this table remain constant over the course of the simulation (i.e., equal to the specified baseline value).

Table 1: Baseline characteristics available to describe the modelled patient population

| **Baseline patient characteristic** | **Mean** | **SE** | **Source** |
| --- | --- | --- | --- |
| Ejection fraction (mL) | 21.00 | 0.18 | PRAISE^1^ |
| Ischemic aetiology (%) | 64.00% | 1.43% |  |
| Systolic blood pressure (mmHg) | 144.58 | 14.46 | Approximated from OPAL-HK CSR; SE taken as 10% of mean^2^ |
| Total cholesterol (mg/dL) | 187.60 | 3.71 | OPAL-HK CSR^2^ |
| Haemoglobin (g/dL) | 13.90 | 0.05 | PRAISE^1^ |
| Percent lymphocytes (%) | 26.00% | 1.31% |  |
| Sodium (mEq/L) | 139.60 | 0.27 | OPAL-HK CSR^2^ |
| Uric acid (mg/dL) | 8.90 | 0.08 | PRAISE^1^ |
| K+ sparing diuretics | 56.29% | 4.04% | Assumption based on OPAL-HK CSR^2^ |
| Beta blocker | 54.30% | 4.05% | OPAL-HK CSR^2^ |
| Statin | 8.00% | 0.81% | PRAISE^1^ |
| Allopurinol | 10.00% | 0.89% |  |
| ICD | 0.00% | 0.00% |  |
| BICD | 0.00% | 0.00% |  |
| Proportion of RAASi users on ACE inhibitors | 67.55% | 3.81% | OPAL-HK CSR^2^ |
| Proportion of RAASi users on ARB | 39.07% | 3.97% |  |
| Diuretic dose (mg/kg) | 1.45 | 0.04 | PRAISE^1^ |
| ACE: angiotensin converting enzyme; ARB: angiotensin II receptor blocker; BICD: biventricular implantable cardioverter defibrillator, BMI: body mass index; ICD: implantable cardioverter defibrillator; RAASi: renin-angiotensin-aldosterone system inhibitor; SE: standard error.  *Note in the OPAL-HK CSR, patients were described only as “stage 4 or worse.”^2^ The proportion of patient’s pre-RRT in stage 5 is thus unknown and here taken as 0. | | | |

## **Natural history evolution of risk factors**

Over the course of the simulation, changes from baseline may be modelled for the following risk factors:

- Age
- RAASi use
- NYHA class
- CKD stage

Age is incremented in line with the model clock (i.e., progression of simulated time). The natural history evolution of CKD and HF are modelled via health state transition probabilities. Changes in RAASi use are modelled based on cyclic probabilities of discontinuation and down-titration stratified by HK event status. Re-initiation of RAASi use may also be modelled.

## **Natural history of CKD and ESRD progression**

The progression through CKD stages and ESRD is modelled according to health state transition probabilities. Progression through CKD stages and ESRD is tracked based on the transition probabilities reported in **Table 2**. The rate of progression to ESRD is demonstrably related to RAASi use and therefore, the ability to apply a hazard ratio to the progression rates from CKD stage 3 to CKD stage 4 and from CKD stage 4 to CKD stage 5 (ESRD), for those receiving RAASi therapy is made available^3^.

Following the initiation of RRT, simulated patients are modelled within an RRT module. Within the RRT module, RRT is broken down into dialysis and transplant health states. Changes in modality of care and the incidence of dialysis-related complications and mortality are controlled via the parameters detailed in **Table 2**.

Calculations used to derive CKD natural history inputs are summarised in **Table 3** to **Table 7**. Additional inputs relating to the calculation of age-dependent ESRD inputs used in RAASi scenario analyses are detailed in **Table 8** and **Table 9.**

Table 2: Model inputs controlling initiation and management of RRT (RRT module)

| **Parameter** | **Mean** | **SE** | **Source** |
| --- | --- | --- | --- |
| **Monthly transition probability** | | | |
| CKD stage 3 to CKD stage 4 | 1.660% | 0.166%* | Nuijten et al.^4^ |
| CKD stage 4 to CKD stage 5 | 0.576% | 0.058%* |  |
| CKD stage 5 to dialysis | 12.640% | 1.614%* | Cooper et al.^5^; time to dialysis of 7.4 months converted to exponential rate and subsequently to probability |
| CKD stage 5 to transplant | 0.176% | 0.018%* | NHSBT^6^ (Table 3.1 and Figure 3.12); Renal Registry - 8^th^ report (Table 5.5)^7^ |
| Dialysis to Transplant | 0.057% | 0.006%* | NHSBT^6^ (Figure 3.13); UK Renal Registry - 23^rd^ report^8^ (Table 2.3); Renal Registry - 8^th^ report (Table 5.5)^7^ |
| Dialysis to Death | 0.846% | 0.085%* | UK Renal Registry – 23^rd^ report^8^ (Table 3.9) |
| Transplant to Dialysis (graft failure) | 0.193% | 0.019%* | NHSBT^6^ (Table 6.1 and 6.2; weighted by values in Figure 5.1); Karim et al.^9^ |
| Transplant to Death | 0.323% | 0.032%* |  |
| **CKD progression odds ratio** | | | |
| On RAASi: CKD stage 3 to CKD stage 4 | 0.61000 | 0.08163 | Xie et al.^3^ |
| On RAASi: CKD stage 4 to CKD stage 5 (ESRD) | 0.61000 | 0.08163 | Xie et al.^3^ |
| eGFR: estimated glomerular filtration rate; HF: heart failure; NHSBT: NHS Blood and Transplant; RAASi: renin–angiotensin–aldosterone system inhibitor; SE: standard error  *SE assumed 10% of mean | | | |

Table 3: Proportion in receipt of peritoneal dialysis or haemodialysis

| **Modality** | **Proportion** | **Source** |
| --- | --- | --- |
| Home HD | 0.021 | Renal Registry (Figure 3.8)^8^ |
| Hospital HD | 0.142 |  |
| Satellite HD | 0.217 |  |
| **Total proportion haemodialysis** | **0.380** |  |
| CAPD | 0.020 | Renal Registry (Figure 3.8)^8^ |
| APD | 0.034 |  |
| **Total proportion peritoneal dialysis** | **0.054** |  |
| **Total proportion transplant** | **0.566** |  |
| APD: automated peritoneal dialysis; CAPD: continuous ambulatory peritoneal dialysis; HD: haemodialysis | | |

Table 4: Annual probability of dialysis complications

| **Parameter** | **HD** | **PD** | **Source** |
| --- | --- | --- | --- |
| **Number of events** | | | |
| MRSA | 80 | 1 | Renal Registry (Table 6.8)^8^ |
| MSSA | 1,271 | 29 |  |
| C.difficile | 498 | 56 |  |
| E.coli | 924 | 67 |  |
| Total | 2,773 | 153 |  |
| **Infection rate per 100 patient years** | | | |
| MRSA | 0.17 | 0.01 | Renal Registry (Table 5.8)^8^ |
| MSSA | 2.72 | 0.43 |  |
| C. difficile | 1.06 | 0.83 |  |
| E. coli | 1.96 | 0.99 |  |
| Sum of rates | 5.91 | 2.26 | Calculated |
| **Distribution** | | | |
| Proportion on modality | 0.876 | 0.124 | See **Table 3**; proportions inflated to sum to 100% |
| **Weighting** | | | |
| Weighted rate per 100 patient years | 5.456 | | Calculated based on weighted distribution across modalities |
| Weighted monthly probability (%) | 0.454% (0.045%)* | |  |
| C. difficile: clostridium difficile; E. coli: Escherichia coli; HD: haemodialysis; MRSA: methicillin-resistant Staphylococcus aureus; MSSA: methicillin-sensitive Staphylococcus aureus  *SE assumed 10% of mean | | | |

Table 5: Monthly probability of dialysis death

| **Parameter** | **Estimate** | **Source** |
| --- | --- | --- |
| **UKRR death rate per 1,000 prevalent RRT patients*** | | |
| Age 40 (age 40-44 2018) | 22 | Renal Registry (Table 5.5)^7^ |
| Age 50 (age 50-54 2018) | 44 | Renal Registry (Table 5.5)^7^ |
| Age 60 (age 60-64 2018) | 74 | Renal Registry (Table 5.5)^7^ |
| Age 65 (age 65-69 2018)** | 102 |  |
| Age 70 (age 70-74 2018) | 148 | Renal Registry (Table 5.5)^7^ |
| *Assumed representative of dialysis deaths and used to calculate the monthly probability of dialysis death using the following formula: Monthly probability of dialysis death = 1 - EXP( -(Death Rate / 1000) / 12)  **Applied in base case; all other estimates are used in RAASi scenario analyses | | |

Table 6: Monthly probability of transplant

| **Parameter** | **Estimate** | **Source** |
| --- | --- | --- |
| **Transplant from CKD stage 5** | | |
| Median waiting time UK (days) | 675 | NHSBT (Table 3.1)^6^ |
| Median waiting time UK (months) | 22.177 | Calculated |
| Proportion of patients pre-emptively listed on transplant waiting list (2017/18) [A] | 38% | NHSBT (Figure 3.12)^6^ |
| Proportion of patients pre-emptively listed on transplant waiting list (age 65+ 2000/01)** [B] | 6% | Renal Registry (Table 5.5)^7^ |
| Proportion of patients pre-emptively listed on transplant waiting list (age 45-54 2000/01)** [C] | 57% | Renal Registry (Table 5.5)^7^ |
| Monthly probability of transplant | 0.176%* | Calculated |
| **Transplant from dialysis** | | |
| Median waiting time UK (days) | 1,088 | NHSBT (Figure 3.13)^6^ |
| Median waiting time UK (months) | 35.745 | Calculated |
| Proportion of patients pre-emptively listed on transplant waiting list [A] | 18.9% | Renal Registry (Table 1.3)^8^ |
| Proportion of patients pre-emptively listed on transplant waiting list (age 65+ 2000/01)** [B] | 6% | Renal Registry (Table 5.5)^7^ |
| Proportion of patients pre-emptively listed on transplant waiting list (age 45-54 2000/01)** [C] | 57% | Renal Registry (Table 5.5)^7^ |
| Monthly probability of transplant | 0.057%* | Calculated |
| CKD: chronic kidney disease  *Calculated as (1-EXP(-(1/median waiting time))) * A * B / C  **Used to approximate age-dependency of transplant operations | | |

Table 7: Annual probability of graft failure and transplant survival

| **Parameter** | **Deceased donor** | **Living donor** | **Source** |
| --- | --- | --- | --- |
| **Graft failure** | | | |
| Five-year probability of graft survival | 87% | 92% | NHSBT (Table 6.1 and 6.2)^6^ |
| Number of patients | 2,339 | 941 | NHSBT (Figure 5.1)^6^ |
| Weighted five-year probability of graft survival | 88.43%* | | Calculated |
| Weighted monthly probability of graft failure | 0.19%† | | Calculated |
| **Patient survival** | | | |
| Five-year probability of patient survival | 87% | 94% | NHSBT (Table 6.1 and 6.2)^6^ |
| Number of patients | 2,339 | 941 | NHSBT (Figure 5.1)^6^ |
| Weighted five-year probability of patient survival | 89.01%* | | Calculated |
| Hazard ratio for mortality post-kidney transplant (age 50-59)** [A] | 2.52 | | Karim et al.^9^ |
| Hazard ratio for mortality post-kidney transplant (age 60-69)** [B] | 4.45 | | Karim et al.^9^ |
| Weighted monthly probability of patient death | 0.18%† | | Calculated |
| NHSBT: national health service blood and transport.  *Calculated as (living donor survival x number of living donor patients + deceased donor survival x number of deceased donor patients) / total number of patients  **Used to approximate age-dependency of transplant survival  †Calculated as 1-EXP(LN((1 - (1 - weighted five-year probability of graft failure) / 60)))  ⸸Calculated as 1-EXP(LN((1 - (1 - weighted five-year probability of graft failure) / 60)) * (B / A)) | | | |

Table 8: Additional transplant input data used to generate age-dependent estimates of transplant for RAASi scenario analyses

| **Parameter** | **Estimate** | **Source** |
| --- | --- | --- |
| **Proportion of patients pre-emptively listed on transplant waiting list*** | | |
| Age 40 (age 35-44 2000/01)** | 73% | Renal Registry (Table 5.5)^7^ |
| Age 50 (age 45-54 2000/01)** | 57% | Renal Registry (Table 5.5)^7^ |
| Age 60 (age 55-64 2000/01)** | 38% | Renal Registry (Table 5.5)^7^ |
| Age 70 (age 65+ 2000/01)** | 6% | Renal Registry (Table 5.5)^7^ |
| *Used in place of [B] in calculations detailed in **Table 6** to calculate the monthly probability of transplant for RAASi scenario analyses | | |

Table 9: Additional transplant survival input data used to generate age-dependent estimates of transplant survival for RAASi scenario analyses

| **Parameter** | **Estimate** | **Source** |
| --- | --- | --- |
| Hazard ratio for mortality post-kidney transplant* | | |
| Age 40 (age <50)** | 1.00 | Karim et al.^9^ |
| Age 50 (age 50-59)** | 2.52 | Karim et al.^9^ |
| Age 60 (age 60-69)** | 4.45 | Karim et al.^9^ |
| Age 70 (age 70+)** | 7.62 | Karim et al.^9^ |
| *Used in place of [B] in calculations detailed in **Table 7** to calculate the monthly probability of transplant survival for RAASi scenario analyses | | |

## **Natural history of HF progression**

Changes in NYHA classification are controlled using monthly probabilities. The model enables the application of different probabilities according to RAASi use (any versus none); however, these are assumed to be equal in the absence of appropriate data (**Table 10**).

Table 10: Monthly probabilities of changes in NYHA classification, with and without RAASi (any use)

|  | **To** | | | | |
| --- | --- | --- | --- | --- | --- |
|  |  | **NHYA I** | **NYHA II** | **NYHA III** | **NYHA IV** |
| **From** | **NHYA I** | 0.7956 | 0.1245 | 0.0738 | 0.0061 |
|  | **NYHA II** | 0.0710 | 0.8448 | 0.0765 | 0.0077 |
|  | **NYHA III** | 0.0047 | 0.0893 | 0.8845 | 0.0216 |
|  | **NYHA IV** | 0.0000 | 0.1064 | 0.1064 | 0.7872 |
| NYHA: New York Heart Association.  Source: Yao et al.^10^  Source publication did not report sufficient data to support sampling of parameters from dirichlet distribution; thus, parameters are sampled independently with SE derived from the number of patients informing the transition probabilities (n=380) | | | | | |

## **RAASi usage inputs**

The calculations for RAASi discontinuation, down-titration and up-titration are presented in **Table 11** and **Table 12**.

Table 11: RAASi discontinuation and down-titration (month 2-3)

|  | **SoC** | | | **Patiromer** | | | **Source** |
| --- | --- | --- | --- | --- | --- | --- | --- |
|  | **No change** | **RAASi dose reduced by 50%** | **RAASi stopped** | **No change** | **RAASi dose reduced by 50%** | **RAASi stopped** |  |
| **Number of RAASi dose reductions and discontinuations** | | | | | | | |
| Week 1 | 44 | 4 | 1 | 53 | 0 | 0 | OPAL-HK CSR^2^ |
| Week 2 | 37 | 6 | 4 | 53 | 0 | 0 |  |
| Week 3 | 37 | 3 | 2 | 50 | 0 | 1 |  |
| Week 4 | 26 | 8 | 5 | 49 | 0 | 0 |  |
| Week 5 | 22 | 3 | 7 | 46 | 0 | 1 |  |
| Week 6 | 24 | 0 | 3 | 46 | 0 | 0 |  |
| Week 7 | 20 | 2 | 1 | 43 | 0 | 1 |  |
| Week 8 | 18 | 1 | 3 | 43 | 0 | 0 |  |
| Total | 228 | 27 | 26 | 383 | 0 | 3 |  |
| Weekly transition probability* | 81.14% | 9.61% | 9.25% | 99.22% | 0.00% | 0.78% | Calculated |
| Monthly transition probability | - | 35.55% | 34.44% | - | 0.00% | 3.34% |  |
| RAASi: renin-angiotensin-aldosterone system inhibitor; SE: standard error; SoC: standard of care  *Calculated as number of transitions observed divide by total number of transitions | | | | | | | |

Table 12: RAASi discontinuation and down-titration (month 4+)

|  | **Potassium threshold: 5.0 mmol/L** | | **Potassium threshold: 5.5 mmol/L** | | **Potassium threshold: 6.0 mmol/L** | | **Source** |
| --- | --- | --- | --- | --- | --- | --- | --- |
|  | **Below threshold** | **Above threshold** | **Below threshold** | **Above threshold** | **Below threshold** | **Above threshold** |  |
| **Dose modification of renin–angiotensin–aldosterone system inhibitor prescriptions ending within 7 days of a serum potassium (K+) measurement, stratified by serum K+ threshold for CKD patients.** | | | | | | | |
| Probability of discontinuation | 2.6% (2.6-2.7%) | 3.7% (3.5-3.9%) | 2.7% (2.6-2.8%) | 5.6% (5.1-6.0%) | 2.8% (2.7-2.9%) | 10.0% (8.7-11.3%) | Linde et al.^11^ |
| Probability of down-titration | 1.8% (1.8-1.9%) | 3.5% (3.3-3.7%) | 2.0% (1.9-2.1%) | 6.0% (5.5-6.4%) | 2.1% (2.1-2.2% | 8.9% (7.6-10.1%) |  |
| Total number of patients | 132,840 | 39,181 | 161,795 | 10,226 | 170,046 | 1,975 |  |
| **Monthly probability of discontinuation and down-titration (K+ <5.0 mmol/L); Mean (SE)** | | | | | | | |
| Discontinuation | 2.600% (0.026%) | | | | | | Linde et al.^11^ |
| Down titration | 1.800% (0.026%) | | | | | |  |
| **Monthly probability of discontinuation and down-titration (K+ 5.0-5.5 mmol/L); Mean (SE)** | | | | | | | |
| Discontinuation | (39181 * 0.037 – 10226 * 0.056) / (39181 - 10226) = 3.029% (0.102%) | | | | | | Calculation (Linde et al.^11^) |
| Down titration | (39181 * 0.035 – 10226 * 0.06) / (39181 - 10226) = 2.617 (0.102%) | | | | | |  |
| **Monthly probability of discontinuation and down-titration (K+ 5.5-6.0 mmol/L); Mean (SE)** | | | | | | | |
| Discontinuation | (10226 * 0.056 – 1975 * 0.1) / (10226 - 1975) = 4.547% (0.230%) | | | | | | Calculation (Linde et al.^11^) |
| Down titration | (10226 * 0.06 – 1975 * 0.089) / (10226 - 1975) = 5.306% (0.230%) | | | | | |  |
| **Monthly probability of discontinuation and down-titration (K+ >6.0 mmol/L); Mean (SE)** | | | | | | | |
| Discontinuation | 10.000% (0.663%) | | | | | | Linde et al.^11^ |
| Down titration | 8.900% (0.638%) | | | | | |  |
| CKD: chronic kidney disease; SE: standard error  Standard errors were calculated by taking the range from the confidence interval and dividing through by 2 and then dividing through by 1.96 | | | | | | | |

## **Incidence of events**

**Table 13** summarises the approach taken to model disease progression and events not directly related to treatment and/or HK incidence (i.e., MACE, hospitalisation, changes in RAASi use and mortality). Data used to inform modelled disease progression and event incidence were primarily sourced from the published literature.

Baseline event rates or probabilities were sourced from published literature for CKD/HF comorbidities where possible; this baseline rate/probability may be modified according RAASi use. Where necessary, the estimated probability of each event is converted to the appropriate cycle length.

Table 13: Summary of methods employed to model disease progression and events

| **Population** | **Events** | **Baseline incidence rate** | **Modified by RAASi use?** | | **Modified by K+ levels?** | |
| --- | --- | --- | --- | --- | --- | --- |
| **CKD** | Progression | Monthly transition probabilities | ✓ | Any versus none | 🗶 |  |
|  | HK | Incidence of K+ above threshold | ✓ | Any versus none | ✓ | By definition |
|  | RAASi discontinuation | By RAASi dose level | ✓✓ | By definition | ✓ | By K+ category |
|  | RAASi down-titration | By RAASi dose level | ✓✓ | By definition | ✓ | By K+ category |
|  | MACE | By CKD stage ** | ✓ | IRR any versus none | ✓ | By K+ category |
|  | Hospitalisation | By CKD stage | 🗶 | Appropriate data not identified | ✓ | By K+ category |
|  | Mortality* | By CKD stage | ✓ | IRR any versus none | ✓ | By K+ category |
| **HF** | Progression (NYHA) | Monthly transition probabilities | 🗶 | Appropriate data not identified | 🗶 |  |
|  | HK | Incidence of K+ above threshold | ✓ | Any versus none | ✓ | By definition |
|  | RAASi discontinuation | By RAASi dose level | ✓✓ | By definition | ✓ | By K+ category |
|  | RAASi down-titration | By RAASi dose level | ✓✓ | By definition | ✓ | By K+ category |
|  | MACE | By RAASi dose level | ✓✓ | Rate for each dose | ✓ | By K+ category |
|  | Hospitalisation | By serum K+ category; CPRD | ✓✓ | OR for each dose versus none | ✓ | By K+ category |
|  | Mortality* | By NYHA class and other risk factors, Seattle Heart Failure Model (SHFM).^1^ | ✓ | Any versus none; Risk factor in SHFM | ✓ | By K+ category |
| CKD: chronic kidney disease; CPRD: Clinical Practice Research Datalink; eGFR: estimated glomerular filtration rate; HK: hyperkalaemia; HR: hazard ratio; IRR: incidence rate ratio, MACE: major adverse cardiac event; OR: odds ratio; SHFM: Seattle Heart Failure Model  RAASi functionality: ✓✓=modified by RAASi use and dose level, ✓=modified by RAASi use (any), 🗶 no functionality  K+ functionality: ✓=functionality 🗶 no functionality  Grey shading indicates events/relationships not modelled due to functionality (🗶) or due to paucity of identified data  *The higher probability based on (A) comorbidity, RAASi use and K+ levels or (B) life tables is applied throughout  **Cardiovascular event defined as hospitalisation for coronary heart disease, heart failure, ischemic stroke, and peripheral arterial disease | | | | | | |

### **CKD-specific risk/probability inputs**

Base case model inputs, relating to the incidence of events that are specific to CKD patients, are presented in **Table 14–Table 16**. It is assumed that the baseline event rates presented in **Table 14** represent risk associated with the reference categories utilised in studies reporting incident rate ratios (IRRs) and odds ratios (ORs) (e.g., normokalaemia or no RAASi use).

Table 14: Baseline MACE, hospitalisation and mortality event rate in CKD patients, by CKD stage

| **Annual event rate mean (SE*)** | **CKD subgroup** | | | **Source** |
| --- | --- | --- | --- | --- |
|  | **3^** | **4** | **5** |  |
| MACE** | 0.0478 (0.0012) | 0.2180 (0.0132) | 0.3660 (0.0450) | Go et al.^12^ |
| Hospitalisation | 0.2141 (0.0021) | 0.8675 (0.0202) | 1.4461 (0.0784) |  |
| Mortality  (all cause) | 0.0157 (0.0007) | 0.1136 (0.0094) | 0.1414 (0.0313) |  |
| CKD: chronic kidney disease; MACE: major adverse cardiac event.  *SE estimated from digitised plot showing 95% confidence intervals  **Cardiovascular events defined in Go et al.^12^ as hospitalisation for coronary heart disease, heart failure, ischemic stroke, and peripheral arterial disease  ^CKD stage 3 event rates derived from weighted average of CKD stage 3a and 3b | | | | |

Table 15: Influence of RAASi on MACE, hospitalisation and mortality event rate in CKD patients

| **Parameter** | **Mean** | **SE** | **Source** |
| --- | --- | --- | --- |
| **Mortality** | | | |
| IRR RAASi vs no RAASi | 0.179† | 0.069* | Linde et al.^11^ |
| **MACE** | | | |
| IRR RAASi vs no RAASi | 0.621† | 0.054* | Linde et al.^11^ |
| **Hospitalisation** | | | |
| IRR RAASi vs no RAASi | 1 | 0 | Null value; no evidence identified |
| CKD: chronic kidney disease; IRR: incidence rate ratio; MACE: major adverse cardiac event; RAASi: renin-angiotensin-aldosterone system inhibitor; SE: standard error  *SE estimated from 95% confidence interval  †Assumes that RAASi use >50% dose versus <50% dose is reflective of RAASi versus no RAASi | | | |

Table 16: Influence of HK on MACE, hospitalisation and mortality event rate in CKD patients

| **Parameter** | **Mean** | **SE*** | **Source** |
| --- | --- | --- | --- |
| **Mortality** | | | |
| IRR Normokalaemia: K+ ≤5 | 1.000 | 0.000 | Luo et al.^13^ |
| IRR K+ >5 to ≤5.5 | 1.140 | 0.056 |  |
| IRR K+ >5.5 to ≤6 | 1.600 | 0.130 |  |
| IRR K+ >6 | 3.310 | 0.464 |  |
| **MACE**** | | | |
| IRR Normokalaemia: K+ ≤5 | 1.000 | 0.000 | Luo et al.^13^ |
| IRR K+ >5 to ≤5.5 | 1.010 | 0.020 |  |
| IRR K+ >5.5 to ≤6 | 1.120 | 0.038 |  |
| IRR K+ >6 | 1.880 | 0.117 |  |
| **Hospitalisation** | | | |
| IRR CKD stage 3 - Normokalaemia: K+ ≤5 | 1.000 | 0.000 | Luo et al.^13^ (CKD stage 3 conservatively assumed as eGFR 40-49mL/min/1.73m^2^ in study) |
| IRR CKD stage 3 - K+ >5 to ≤5.5 | 1.070 | 0.089 |  |
| IRR CKD stage 3 - K+ >5.5 to ≤6 | 1.230 | 0.179 |  |
| IRR CKD stage 3 - K+ >6 | 1.910 | 0.564 |  |
| IRR CKD stage 4/5 - Normokalaemia: K+ ≤5 | 1.000 | 0.000 |  |
| IRR CKD stage 4/5 - K+ >5 to ≤5.5 | 1.000 | 0.102 |  |
| IRR CKD stage 4/5 - K+ >5.5 to ≤6 | 1.340 | 0.179 |  |
| IRR CKD stage 4/5 - K+ >6 | 3.650 | 0.584 |  |
| CKD: chronic kidney disease; IRR: incidence rate ratio; MACE: major adverse cardiac event; RAASi: renin-angiotensin-aldosterone system inhibitor; SE: standard error  *SE estimated from 95% confidence interval | | | |

### **HF-specific risk/probability inputs**

Base case model inputs, relating to the incidence of events that are specific to HF patients, are presented in **Table 17-Table 19**. It is assumed that the baseline event rates presented in **Table 17** represent risk associated with the reference categories utilised in studies reporting incident rate ratios (IRRs) and odds ratios (ORs) (e.g., normokalaemia or no RAASi use).

Table 17: Baseline MACE event rates and hospitalisation probabilities in HF patients

| **Parameter** | **Mean** | **SE*** | **Source** |
| --- | --- | --- | --- |
| **Annual MACE event rate** | | | |
| Discontinued RAASi | 0.2904 | 0.0040***** | Assumed as RAASi sub-max |
| RAASi max | 0.1485 | 0.0030***** | Linde et al.^11^ |
| RAASi sub-max | 0.2904 | 0.0040***** |  |
| **Monthly probability of hospitalisation** | | | |
| NYHA I | 0.0152 | 0.0015** | Ford et al.^14^ |
| NYHA II | 0.0240 | 0.0024** |  |
| NYHA III | 0.0240 | 0.0024** |  |
| NYHA IV | 0.1540 | 0.0154** |  |
| MACE: major adverse cardiac event; NYHA; New York Heart Association stage; RAASi: renin-angiotensin-aldosterone system inhibitor; SE: standard error  *SE estimated from 95% confidence intervals  **SE assumed as 10% of mean | | | |

Table 18: Influence of RAASi on hospitalisation in HF patients

| **Parameter** | **Mean** | **SE** | **Source** |
| --- | --- | --- | --- |
| **Hospitalisation** | | | |
| OR RAASi vs no RAASi | 0.670 | 0.033 | Flather et al.^15^ |
| OR Sub RAASi vs no RAASi | 0.835 | 0.033 | Assumption† |
| HF: heart failure; OR: odds ratio; RAASi: renin-angiotensin-aldosterone system inhibitor; SE: standard error  †Mean assumed to be 50% impact of maximum dose RAASi, with equal SE | | | |

Table 19: Influence of HK on MACE, hospitalisation and mortality event rate in CKD patients

| **Parameter** | **Mean** | **SE*** | **Source** |
| --- | --- | --- | --- |
| **Mortality** | | | |
| HR Normokalaemia: K+ ≤5 | 1.000 | 0.000 | Krogager et al.^16^ |
| HR K+ >5 to ≤5.5 | 1.290 | 0.318 |  |
| HR K+ >5.5 to ≤6 | 3.613 | 0.973 |  |
| HR K+ >6 | 3.613 | 0.973 |  |
| **MACE**** | | | |
| IRR Normokalaemia: K+ ≤5 | 1.000 | 0.000 | Assumed as for CKD patients (Luo et al.^13^) |
| IRR K+ >5 to ≤5.5 | 1.010 | 0.020 |  |
| IRR K+ >5.5 to ≤6 | 1.120 | 0.038 |  |
| IRR K+ >6 | 1.880 | 0.117 |  |
| **Hospitalisation** | | | |
| IRR CKD stage 3 - Normokalaemia: K+ ≤5 | 1.000 | 0.000 | Assumed as for CKD patients (Luo et al.^13^) |
| IRR CKD stage 3 - K+ >5 to ≤5.5 | 1.070 | 0.089 |  |
| IRR CKD stage 3 - K+ >5.5 to ≤6 | 1.230 | 0.179 |  |
| IRR CKD stage 3 - K+ >6 | 1.910 | 0.564 |  |
| IRR CKD stage 4/5 - Normokalaemia: K+ ≤5 | 1.000 | 0.000 |  |
| IRR CKD stage 4/5 - K+ >5 to ≤5.5 | 1.000 | 0.102 |  |
| IRR CKD stage 4/5 - K+ >5.5 to ≤6 | 1.340 | 0.179 |  |
| IRR CKD stage 4/5 - K+ >6 | 3.650 | 0.584 |  |
| CKD: chronic kidney disease; HR: hazard ratio; IRR: incidence rate ratio; MACE: major adverse cardiac event; OR: odds ratio; RAASi: renin-angiotensin-aldosterone system inhibitor; SE: standard error  *SE estimated from 95% confidence interval | | | |

**Seattle Heart Failure Model**

Mortality in the HF population is modelled via implementation of the SHFM^1^: a multivariate Cox model for survival among HF patients. Coefficient estimates of the SHFM are presented in **Table 20**. Since the use of ACE inhibitors and ARBs are among the predictive factors of the SHFM, the proportion of RAASi users that are on ACE and ARB therapy are included in the baseline characteristics.

Table 20: SHFM for survival in HF patients^1^

| **Explanatory variable** | **Hazard ratio** | | |
| --- | --- | --- | --- |
|  | **Mean** | **95% CI** | **SE*** |
| Age (years/10) | 1.09 | (0.985–1.205) | 0.0561 |
| Male sex | 1.089 | (0.839–1.414) | 0.1467 |
| NYHA (1–4) | 1.6 | (1.019–2.511) | 0.3806 |
| 100/Ejection fraction | 1.03 | (1.010–1.050) | 0.0102 |
| Ischemic aetiology (0/1) | 1.354 | (1.074–1.707) | 0.1615 |
| SBP (mmHg/10) | 0.877 | (0.823–0.935) | 0.0286 |
| Diuretic dose (mg/kg) | 1.178 | (1.097–1.266) | 0.0431 |
| Allopurinol use (0/1) | 1.571 | (1.170–2.109) | 0.2395 |
| Statin use (0/1) | 0.63 | (0.410–0.978) | 0.1449 |
| If sodium<138, 138-sodium | 1.05 | (1.005–1.097) | 0.0235 |
| Cholesterol (100/mg/dL) | 2.206 | (1.045–4.656) | 0.9212 |
| If haemoglobin <16, 16-haemoglobin | 1.124 | (1.053–1.200) | 0.0375 |
| If haemoglobin >16, haemoglobin-16 | 1.336 | (1.010–1.767) | 0.1931 |
| Lymphocytes (%/5) | 0.897 | (0.846–0.951) | 0.0523 |
| Uric acid (mg/dL) | 1.064 | (1.022–1.108) | 0.0219 |
| ACE use (0/1) | 0.77 | - | 0.0770 |
| Beta blocker use (0/1) | 0.66 | - | 0.0660 |
| ARB use (0/1) | 0.85 | - | 0.0850 |
| K-sparing diuretic use (0/1) | 0.74 | - | 0.0740 |
| ICD (0/1) | 0.73 | - | 0.0730 |
| BICD (0/1) | 0.79 | - | 0.0790 |
| ACE: angiotensin converting enzyme; ARB: angiotensin receptor blocker; BICD: Biventricular implantable cardioverter-defibrillator; NYHA: New York Heart Association classification; ICD: Implantable cardioverter-defibrillator  *SE estimated from 95% confidence interval, or assumed as 10% of mean where CI not presented | | | |

### **Life tables**

Life tables are utilised to describe all-cause mortality experienced in the general population, where each row contains the probability that an individual aged x will die by age x+1. National life tables are typically available in this form, or similar, up to age 100. After this age, individuals are assumed to survive no longer; in other words, modelled individuals that survive to age 101 die in the next modelled cycle (**Table 21**).

Table 21: Excerpt from 2015-2017 Irish life tables

| **Age** | **Annual probability of death** | |
| --- | --- | --- |
|  | **Male** | **Female** |
| 65 | 0.011 | 0.007 |
| 66 | 0.012 | 0.008 |
| 67 | 0.014 | 0.009 |
| ... | ... | … |
| 98 | 0.265 | 0.301 |
| 99 | 0.267 | 0.318 |
| 100 | 0.270 | 0.334 |
| *101* | *1.00* | *1.00* |
| Source: Ireland Central Statistics Office^17^ | | |

## **Treatment duration, discontinuation and retreatment**

Modelled patients receive treatment (Patiromer) from the beginning of the simulation until one of the following occurs:

1. Patient reaches ESRD and commences RRT
2. Modelled K+ level falls below a user-defined acceptable range for discontinuation of treatment
3. Discontinuation due to other reasons, defined by a monthly probability of discontinuation

Following discontinuation due to any reason, patients incur HK and RAASi discontinuation/down-titration risk in line with the SoC arm. SoC may not be discontinued.

The model also simulates repeat treatment in patients that discontinue Patiromer due to reasons B or C above. Repeat treatment is initiated when the patient’s modelled K+ level rises above 5.5 mmol/L. If treatment is repeated, K+ is modelled according to the patiromer profile specified for the first month of treatment.

**Figure 1** presents the logical process followed to simulate treatment discontinuation (patiromer) and repeat treatment.


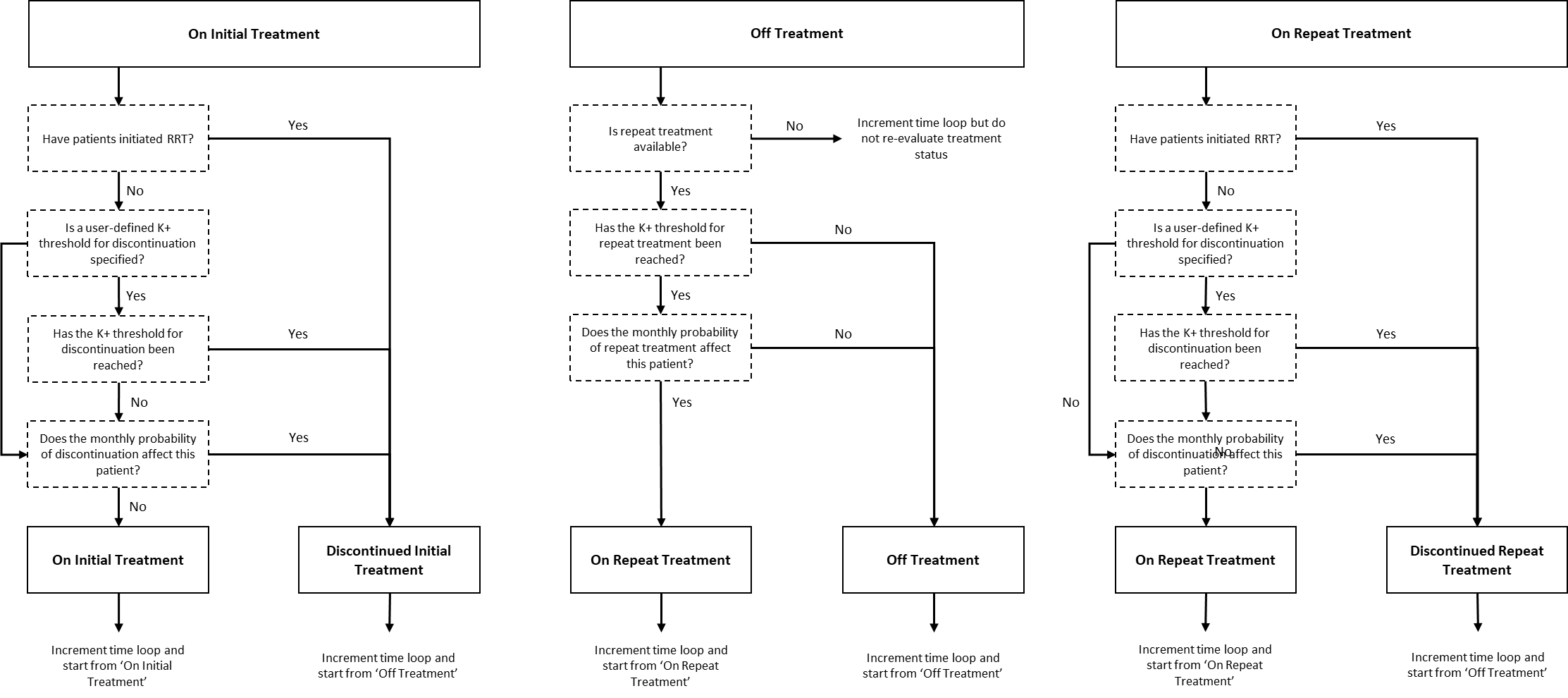


Figure 1: Treatment discontinuation and re-initiation algorithm (individual patient perspective)

### **K+ threshold for repeat treatment**

Discontinued patients may initiate repeat treatment after the first cycle if their modelled K+ value rises above 5.5 mmol/L (prior to the initiation of RRT). Repeat treatment corresponds to the initial patiromer treatment; however, in a simplified form, only the input values relating to month 1 of initial treatment are used (i.e., treatment response after 1 month, RAASi status after 1 month and HK event rates during the first month of treatment). The K+ threshold for repeat treatment is applied inclusive of the threshold value, such that repeat treatment is modelled if K+ equals or exceeds the threshold value. Repeat treatment may occur multiple times.

### **Monthly probability of discontinuation**

Modelled patients are subject to a monthly probability of discontinuing treatment. Monthly discontinuation probabilities for patiromer were estimated using available data, as summarised in **Table 22**. Since SoC comprises a combination of lifestyle interventions and routine HK management, the probability of discontinuation from SoC is assumed to be 0. For Patiromer, the annual probability of discontinuation was estimated using data from the extended phase of the OPAL-HK trial^2^.

Table 22: Monthly probability of discontinuation

|  | **Mean (%)** | **SE (%)** | **Source** |
| --- | --- | --- | --- |
| Patiromer | 10.33* | 4.10 | OPAL-HK CSR^2^ |
| * Extended phase probability, with 10/55 patients discontinuing during the 8-week period, adjusted to monthly probability | | | |

## **Notes on the application of odds ratios and hazard ratios**

**Odds ratios (ORs)**

Where ORs are applied in the model, for consistency they are applied to annual probabilities. Probabilities are converted to odds using the formula: odds = probability / (1 – probability). The odds ratio is subsequently multiplied by the odds, and the resultant odds is converted back to a probability using the formula: probability = odds / (1 + odds).

The resultant probability may then be converted to the necessary time frame (e.g., monthly cycle).

**Hazard ratios (HRs)**

Hazard ratios are applied directly to rates. If inputs are loaded as probabilities, they are converted to rates before applying the hazard ratio and are then converted back to probabilities.

**Probability / rate conversion**

Probabilities are converted to rates using the following formula: p = 1 – Exp(- r * t)

Rates are converted to probabilities using the following formula: r = -ln(1 – p) / t

In both the above, p = probability; r = rate; t = time.

## **References**

1. Levy WC, Mozaffarian D, Linker DT, et al. The Seattle Heart Failure Model prediction of survival in heart failure. Circulation. 2006;113(11):1424-33.

2. Vifor Pharma. OPAL-HK CSR. Data on file. 2014.

3. Xie X, Liu Y, Perkovic V, et al. Renin-angiotensin system inhibitors and kidney and cardiovascular outcomes in patients with CKD: a Bayesian network meta-analysis of randomized clinical trials. American Journal of Kidney Diseases. 2016;67(5):728-41.

4. Nuijten M, Andress DL, Marx SE, et al. Cost Effectiveness of Paricalcitol versus a non-selective vitamin D receptor activator for secondary hyperparathyroidism in the UK: a chronic kidney disease markov model. Clin Drug Investig. 2010;30(8):545-57.

5. Cooper BA, Branley P, Bulfone L, et al. A randomized, controlled trial of early versus late initiation of dialysis. N Engl J Med. 2010;363(7):609-19.

6. NHS Blood and Transport. Annual report on kidney transplantation. 2019.

7. UK Renal Association. UK Renal Registry 8th Annual Report 2004. . 2004. Available at: <https://renal.org/about-us/who-we-are/uk-renal-registry> [Accessed February 2021].

8. UK Renal Association. UK Renal Registry 23rd Annual Report 2021. . 2021. Available at: <https://renal.org/about-us/who-we-are/uk-renal-registry> [Accessed February 2021].

9. Karim A, Farrugia D, Cheshire J, et al. Recipient Age and Risk for Mortality After Kidney Transplantation in England. Transplantation. 2014;97(8):832-8.

10. Yao G, Freemantle N, Calvert MJ, et al. The long-term cost-effectiveness of cardiac resynchronization therapy with or without an implantable cardioverter-defibrillator. European heart journal. 2007;28(1):42-51.

11. Linde C, Bakhai A, Furuland H, et al. Real-World Associations of Renin-Angiotensin-Aldosterone System Inhibitor Dose, Hyperkalemia, and Adverse Clinical Outcomes in a Cohort of Patients With New-Onset Chronic Kidney Disease or Heart Failure in the United Kingdom. J Am Heart Assoc. 2019;8(22):e012655.

12. Go AS, Chertow GM, Fan D, et al. Chronic kidney disease and the risks of death, cardiovascular events, and hospitalization. New England Journal of Medicine. 2004;351(13):1296-305.

13. Luo J, Brunelli SM, Jensen DE, et al. Association between serum potassium and outcomes in patients with reduced kidney function. Clin J Am Soc Nephrol. 2016;11(1):90-100.

14. Ford E, Adams J, Graves N. Development of an economic model to assess the cost-effectiveness of hawthorn extract as an adjunct treatment for heart failure in Australia. BMJ Open. 2012;2(5):e001094.

15. Flather MD, Yusuf S, Køber L, et al. Long-term ACE-inhibitor therapy in patients with heart failure or left-ventricular dysfunction: a systematic overview of data from individual patients. ACE-Inhibitor Myocardial Infarction Collaborative Group. Lancet. 2000;355(9215):1575-81.

16. Krogager ML, Eggers-Kaas L, Aasbjerg K, et al. Short-term mortality risk of serum potassium levels in acute heart failure following myocardial infarction. European Heart Journal-Cardiovascular Pharmacotherapy. 2015:pvv026.

17. Central Statistics Office. Irish Life Tables 2015-2017. 2020.
