# Supplementary material for: A cost-effectiveness analysis of patiromer in the UK: evaluation of hyperkalaemia treatment and lifelong RAASi maintenance in chronic kidney disease patients with and without heart failure
Source: BMC Nephrol. 2023 Mar 9;24:47. doi: 10.1186/s12882-023-03088-3 (PMC9995261; doi:10.1186/s12882-023-03088-3)
Supplement: Supplementary file 2 — Additional file 2: This appendix provides details of cost data utilised in the model. [file 12882_2023_3088_MOESM2_ESM.docx]

**Supplemental Appendix B**

This appendix provides details of cost data utilised in the model. All costs are presented in 2019-2020 GBP.

1. **Health state and event costs**

**Table 1** summarises the direct costs applied to modelled health states and events. **Table 2-Table 6** present further details of the derivation of the cost input values associated with renal replacement therapy. Where more than one event can occur within a patient’s lifetime (e.g., hospitalisation events or MACE), the same costs are applied to initial and subsequent events.

Table 1: Health state and event costs

| **Parameter** | **Mean (£)** | **SE*** | **Source** |
| --- | --- | --- | --- |
| Annual cost CKD 3 | 3,690.40 | 369.04* | NICE CG182^1^ † |
| Annual cost CKD 4 | 3,690.40 | 369.04* | NICE CG182^1^ † |
| Annual cost CKD 5 (pre-RRT) | 5,757.75 | 575.77* | NICE CG182^1^ † |
| Annual cost of dialysis | 37,364.01 | 3,736.40* | Baboolal et al.^2,^ † |
| Dialysis access cost | 2,164.24 | 216.42* | NHS ref costs^3^ (Weighted by dialysis access modality) † |
| One-off cost of dialysis complications | 6,257.07 | 625.71* | NICE CG125 B ^4^  (Weighted by dialysis access modality) † |
| One-off transplant procedure cost | 9,263.62 | 926.36* | Weighted NHS ref costs^3^† |
| One-off Organ Transplantation Service cost | 17,356.26 | 1,735.63* | Appendix G† |
| Annual cost of transplant maintenance | 7,212.94 | 721.29* | NICE CG125^4^ |
| NYHAI | 0.00 | 0.00 | Assumption |
| NYHAII | 0.00 | 0.00 | Assumption |
| NYHAIII | 0.00 | 0.00 | Assumption |
| NYHAIV | 0.00 | 0.00 | Assumption |
| Event cost: MACE | 5,205.14 | 520.51* | Kent et al.^5^ |
| Event cost: Hospitalisation | 2,650.41 | 265.04* | Colquitt et al.^6^ |
| Event cost: RAASi discontinuation | 495.63 | 49.56* | **Table 9** |
| Event cost: RAASi down-titration | 743.44 | 74.34* | **Table 7** |
| Event cost: return to maximum RAASi use | 136.69 | 13.67* | **Table 8** |
| CKD: chronic kidney disease; MACE, Major adverse cardiac event; NHYA: New York Heart Association; RRT: renal replacement therapy.  *SE values assumed 10% of the mean. | | | |

**RRT costs**

Renal replacement therapy calculations are summarised in **Table 2-Table 6**.

Dialysis access costs were stratified by dialysis type, with the final cost derived from a weighted average of the two (**Table 2**).

Typically, dialysis can be stratified into four core modalities: hospital haemodialysis, satellite haemodialysis, home dialysis and peritoneal dialysis. Cost data for each modality was identified and weighted in line with the observed patient usage in 2018/19 based on the UK Renal Registry (**Table 3**).

Costs for dialysis complications are described in **Table 4** and are derived from a previous NICE guideline. Transplant costs are described in **Table 5** and **Table 6,** and are derived from NHS Reference costings.

Table 2: Dialysis access cost

|  | **Proportion use** | **Unit Cost (2018/19)** | **Unit cost (2019/20)** | **Weighted average cost** |
| --- | --- | --- | --- | --- |
| Source | See Supplemental Appendix A | NHS ref costs^3^ | Table 21 | - |
| Peritoneal dialysis | 0.122 | £844.92 | £863.59 | £105.27 |
| Haemodialysis | 0.878 | £2,294.09 | £2,344.79 | £2,058.97 |
| **Total weighted average cost of dialysis access** |  |  |  | **£2,164.24** |
| Renal replacement cost for peritoneal dialysis based on NHS references costs 2018-19, currency code LA05Z: Renal replacement peritoneal dialysis associated procedures  Renal replacement cost for haemodialysis based on NHS references costs 2018-19, currency code YQ42Z: open arteriovenous fistula, graft or shunt procedure. | | | | |

Table 3: Annual cost of dialysis

|  | **Proportion use** | **Unit Cost**  **(2008/09)** | **Unit Cost (2019/20)** | **Weighted average cost** |
| --- | --- | --- | --- | --- |
| Source | Renal registry | Baboolal et al.^2^ | Table 21 | - |
| Home HD | 0.045 | £20,764.00 | £24,710.73 | £1,115.61 |
| Hospital HD | 0.375 | £35,023.00 | £41,680.01 | £15,618.24 |
| Satellite HD | 0.458 | £32,669.00 | £38,878.58 | £17,815.69 |
| CAPD | 0.045 | £15,570.00 | £18,529.48 | £836.55 |
| APD | 0.077 | £21,655.00 | £25,771.09 | £1,977.92 |
| **Total weighted average cost** | | | | **£37,364.01** |
| APD: automated peritoneal dialysis; CAPD: continuous ambulatory peritoneal dialysis; HD: haemodialysis**.**  **Note:** The value presented for ‘*Total weighted average cost’* has been scaled to be representative of the proportions presented by dialysis modality. | | | | |

Table 4: Cost of dialysis complications (one off)

|  | **Weighting** | **Unit Cost (2008/09)** | **Unit cost (2019/20)** | **Weighted average cost** |
| --- | --- | --- | --- | --- |
| Source | CG125^4^ | CG125^4^ | Table 21 |  |
| Peritoneal dialysis | 0.122 | £3,103.00 | £3,692.80 | £450.14 |
| Haemodialysis | 0.878 | £5,062.00 | £6,613.04 | £5,806.93 |
| **Total weighted average cost** | | | | **£6,257.07** |
| **Note:** The value presented for ‘*Total weighted average cost’* has been scaled to be representative of the proportions presented by dialysis modality. | | | | |

Table 5: Cost of transplant, procedural cost

| **Currency code** | **Currency description** | **Activity** | **Unit cost (2018/19)** | **Unit cost (2019/20)** | **Weighted cost** |
| --- | --- | --- | --- | --- | --- |
| LA01A | Kidney Transplant, 19 years and over, from cadaver non-heart beating donor | 772 | £12,605.44 | £12,884.02 | £3,709.98 |
| LA02A | Kidney Transplant, 19 years and over, from cadaver heart beating donor | 1,196 | £12,988.82 | £13,275.87 | £5,922.40 |
| LA03A | Kidney Transplant, 19 years and over, from live donor | 713 | £12,291.90 | £12,563.55 | £3,341.22 |
| **Weighted average cost** | |  |  |  | **£12,973.60** |
| All activity and unit costs were sourced from NHS reference costs 2018-19^3^ | | | | | |

Table 6: Cost of transplant, organ transplantation services

| **No of transplants performed** | **Annual expenditure (£)** | **Cost per transplant (2011/12)** | **Cost per transplant (2019/20)** |
| --- | --- | --- | --- |
| 3,960 | £61,106,000 | £15,430.81 | £17,356.26 |
| Note: Costs associated with organ donation and transplant activities (2011-12) were obtained by Freedom of Information request. The annual expenditure was divided by the number of transplants performed in 2011/12 to derive a cost per transplant, which was then inflated to 2019-20 prices. | | | |

**RAASi discontinuation costs**

The derivation of RAASi discontinuation costs is detailed in **Table 7** to **Table 9.**

Table 7. RAASi dose down-titration

| **Resource** | **Proportion use (%)** | **Resource use** | **Unit Cost** | **Source** | **Total cost** |
| --- | --- | --- | --- | --- | --- |
| **Primary care** | **50.00** |  | | | |
| GP visit | 100.00 | 3.00 | £39.00 | PSSRU 2020 ^7^ | £117.00 |
| U&E test | 100.00 | 3.00 | £6.56 | NICE guidelines [NG45] ^8^ | £19.69 |
| **Total primary care costs** | | | | | **£136.69** |
| **Secondary care costs** | **50.00** |  | | | |
| Outpatient visit | 50.00 | 3.00 | £135.00 | PSSRU 2020 ^7^ | £202.50 |
| Inpatient day | 50.00 | 3.00 | £752.00 | PSSRU 2020 ^7^ | £1,128.00 |
| U&E test | 100.00 | 3.00 | £6.56 | NICE guidelines [NG45] ^8^ | £19.69 |
| **Total Secondary care costs** | | | | | **£1,350.19** |
| **Weighted RAASi dose down-titration costs** | | | | | **£743.44** |
| GP: general practitioner; RAASi: renin–angiotensin–aldosterone system inhibitor; U&E: urea and electrolytes | | | | | |

Table 8. RAASi dose up-titration cost

| **Resource** | **Proportion use (%)** | **Resource use** | **Unit Cost** | **Source** | **Total cost** |
| --- | --- | --- | --- | --- | --- |
| **Primary care** | **100.00** |  | | | |
| GP visit | 100.00 | 3.00 | £39.00 | PSSRU 2020 ^7^ | £117.00 |
| U&E test | 100.00 | 3.00 | £6.56 | NICE guidelines [NG45] ^8^ | £19.69 |
| **Total primary care costs** | | | | | **£136.69** |
| **Secondary care costs** | **0.00** |  | | | |
| Outpatient visit | 0.00 | 0.00 | £135.00 | PSSRU 2020 ^7^ | £202.50 |
| Inpatient day | 0.00 | 0.00 | £752.00 | PSSRU 2020 ^7^ | £1,128.00 |
| U&E test | 0.00 | 0.00 | £6.56 | NICE guidelines [NG45] ^8^ | £19.69 |
| **Total Secondary care costs** | | | | | **£0.00** |
| **Weighted RAASi dose up-titration costs** | | | | | **£136.69** |
| GP: general practitioner; RAASi: renin–angiotensin–aldosterone system inhibitor; U&E: urea and electrolytes | | | | | |

Table 9. RAASi discontinuation cost

| **Resource** | **Proportion use (%)** | **Resource use** | **Unit Cost** | **Source** | **Total cost** |
| --- | --- | --- | --- | --- | --- |
| **Primary care** | **50.00** |  | | | |
| GP visit | 100.00 | 2.00 | £39.00 | PSSRU 2020 ^7^ | £78.00 |
| U&E test | 100.00 | 2.00 | £6.56 | NICE guidelines [NG45] ^8^ | £13.13 |
| **Total primary care costs** | | | | | **£91.13** |
| **Secondary care costs** | **50.00** |  | | | |
| Outpatient visit | 50.00 | 2.00 | £135.00 | PSSRU 2020 ^7^ | £135.00 |
| Inpatient day | 50.00 | 2.00 | £752.00 | PSSRU 2020 ^7^ | £752.00 |
| U&E test | 100.00 | 2.00 | £6.56 | NICE guidelines [NG45] ^8^ | £13.13 |
| **Total Secondary care costs** | | | | | **£900.13** |
| **Weighted RAASi discontinuation costs** | | | | | **£495.63** |
| GP: general practitioner; RAASi: renin–angiotensin–aldosterone system inhibitor; U&E: urea and electrolytes | | | | | |

1. **RAASi therapy costs**

Costs associated with ongoing RAASi therapy (ACE, ARB and/or mineralocorticoid receptor agonist [MRA]) are applied at two levels, corresponding to “max” and “sub-max” levels referenced elsewhere. Annual costs for the two categories are derived from a series of user-defined inputs describing the proportions of patients receiving each therapy and average doses under “max” and “sub-max” therapy. These are combined with unit costs per mg, to derive weighted average costs per patient receiving RAASi therapy at each level.

**Table 10** summarises annual aggregated RAASi costs, whilst **Table 11** and **Table 12** describe their derivation in more detail.

Table 10: Costs applied to RAASi use

| **Parameter** | **Mean (£)** | **SE** | **Source** |
| --- | --- | --- | --- |
| Annual cost of RAASi: Optimal therapy (Max) | 33.48 | 3.35 | See Table 11 and Table 12 PHS drug tariffs part 7 (1154711000001107)^9^ |
| Annual cost of RAASi: Sub-optimal therapy (Sub-max) | 16.74 | 1.67 |  |

Table 11: Weighted annual RAASi cost – Optimal therapy (PHS drug tariff costs)

| **Agent** | **Percentage of cohort** | **Source** | **Average daily dose (mg)** | **Source** | **Cost per mg** | **PHS drug code** |
| --- | --- | --- | --- | --- | --- | --- |
| **ACE inhibitor** | | | | | | |
| Ramipril | 67.5% | OPAL-HK^10^; ramipril assumed representative | 10.00 | ESC^11^ | £0.0074 | 1069111000001107, 5011311000001105 |
| Lisinopril | 0.0% |  | 27.50 | ESC^11^ | £0.0020 | 1247111000001100 |
| Perindopril erbumine | 0.0% |  | 4.00 | MIMS^12^ | £0.0333 | 1096511000001105 |
| Enalapril | 0.0% |  | 40.00 | ESC^11^ | £0.0180 | 988511000001101 |
| Lisinopril / Hydrochlorothiazide | 0.0% |  | 22.50 | MIMS^12^ | £0.0026 | 3143011000001101 |
| Captopril | 0.0% |  | 150.00 | ESC^11^ | £0.0009 | 1040911000001109 |
| **ARB** | | | | | | |
| Candesartan | 39.1% | OPAL-HK^10^; candesartan assumed representative | 32.00 | ESC^11^ | £0.0043 | 8983811000001102 |
| Losartan | 0.0% |  | 150.00 | ESC^11^ | £0.0011 | 988311000001107 |
| Irbesartan | 0.0% |  | 225.00 | MIMS^12^ | £0.0013 | 979211000001102 |
| Valsartan | 0.0% |  | 320.00 | ESC^11^ | £0.0027 | 1272411000001108 |
| Olmesartan medoxomil | 0.0% |  | 30.00 | MIMS^12^ | £0.0181 | 4624211000001106, 4624511000001109 |
| Telmisartan | 0.0% |  | 50.00 | MIMS^12^ | £0.0056 | 1102911000001106 |
| **MRA** | | | | | | |
| Spironolactone BP | 7.3% | OPAL-HK^10^; spironolactone assumed representative | 50.00 | ESC^11^ | £0.0037 | 951511000001102 |
| Eplerenone | 0.0% |  | 50.00 | ESC^11^ | £0.0224 | 8479611000001107 |
| **Weighted average (daily) daily cost** | **£0.0917** | | | | | |
| **Weighted average annual cost** | **£33.48** | | | | | |
| ACE: angiotensin converting enzyme; ARB: Angiotensin II Receptor Blockers; CKD: Chronic Kidney Disease; HF: Heart Failure; MRA: Mineralcorticoid Receptor Antagonist; RAASi: renin–angiotensin–aldosterone system inhibitor. Public health Scotland Scottish Drug Tariff part 7 (Mar 2021)^9^ Weighted average daily cost: Average daily dose (mg) x Cost per mg x Percentage of cohort on Drug; Weighted average annual cost: Weighted average daily cost x 365.25 | | | | | | |

Table 12: Weighted annual RAASi cost – Sub-optimal dosing

| **Agent** | **Percentage of cohort** | **Source** | **Average daily dose (mg)** | **Source** | **Cost per mg** | **PHS drug code** |
| --- | --- | --- | --- | --- | --- | --- |
| **ACE inhibitor** | | | | | | |
| Ramipril | 67.5% | OPAL-HK^10^; ramipril assumed representative | 5.00 | ESC^11^ | £0.0074 | 1069111000001107, 5011311000001105 |
| Lisinopril | 0.0% |  | **-** | ESC^11^ | £0.0020 | 1247111000001100 |
| Perindopril erbumine | 0.0% |  | **-** | MIMS^12^ | £0.0333 | 1096511000001105 |
| Enalapril | 0.0% |  | **-** | ESC^11^ | £0.0180 | 988511000001101 |
| Lisinopril / Hydrochlorothiazide | 0.0% |  | **-** | MIMS^12^ | £0.0026 | 3143011000001101 |
| Captopril | 0.0% |  | **-** | ESC^11^ | £0.0009 | 1040911000001109 |
| **ARB** | | | | | | |
| Candesartan | 39.1% | OPAL-HK^10^; candesartan assumed representative | 16.00 | ESC^11^ | £0.0043 | 8983811000001102 |
| Losartan | 0.0% |  | **-** | ESC^11^ | £0.0011 | 988311000001107 |
| Irbesartan | 0.0% |  | **-** | MIMS^12^ | £0.0013 | 979211000001102 |
| Valsartan | 0.0% |  | **-** | ESC^11^ | £0.0027 | 1272411000001108 |
| Olmesartan medoxomil | 0.0% |  | **-** | MIMS^12^ | £0.0181 | 4624211000001106, 4624511000001109 |
| Telmisartan | 0.0% |  | **-** | MIMS^12^ | £0.0056 | 1102911000001106 |
| **MRA** | | | | | | |
| Spironolactone BP | 7.3% | OPAL-HK^10^; spironolactone assumed representative | 25.00 | ESC^11^ | £0.0037 | 951511000001102 |
| Eplerenone | 0.0% |  | **-** | ESC^11^ | £0.0224 | 8479611000001107 |
| **Weighted average (daily) daily cost** | **£0.0458** | | | | | |
| **Weighted average annual cost** | **£16.74** | | | | | |
| ACE: angiotensin converting enzyme; ARB: Angiotensin II Receptor Blockers; CKD: Chronic Kidney Disease; HF: Heart Failure; MRA: Mineralcorticoid Receptor Antagonist; RAASi: renin–angiotensin–aldosterone system inhibitor. Public health Scotland Scottish Drug Tariff part 7 (Mar 2021)^9^ Weighted average daily cost: Average daily dose (mg) x Cost per mg x Percentage of cohort on Drug; Weighted average annual cost: Weighted average daily cost x 365.25 Note average cost per mg is numerically determined as in Table 11 rather than using different dose strength as it is unknown how in a clinical context how the sub-optimal dose may be prescribed. | | | | | | |

1. **HK treatment costs**

Costs associated with the management of HK events in the maintenance phase have been defined for two K+ thresholds: K+ >5.5 to ≤6 mmol/L and K+ >6.0 mmol/L. These costs are based on expected resource use for HK at each level and are applied during the month of incidence only. The cost inputs are summarised in **Table 13** (see **Table 16** to **Table 18** for a detailed derivation) and are based on those used in NICE TA599^13^.

Table 13. HK event costs

| **Resource** | **Patiromer** | **SoC** | **Source** |
| --- | --- | --- | --- |
| HK event: K+ >5.5 to ≤6 mmol/L | 223.11 (22.31) | 223.11 (22.31) | **Table 16** to **Table 18** |
| HK event: K+ >6.0 mmol/L | 2,943.38 (294.34) | 2,933.49 (293.35) | **Table 16** to **Table 18** |
| HK: hyperkalaemia; SE: standard error; SoC: standard of care  *SE assumed as 10% of mean | | | |

Table 16. HK event cost: K+ ≥6 mEq/L

| **Resource** | **Percentage (%)** | **Unit** | **Unit Cost** | **Source** | **Total cost** |
| --- | --- | --- | --- | --- | --- |
| **Inpatients diagnosis** | **100.00** |  | | | |
| Inpatient day | 100.00 | 1.00 | £602.00 | PSSRU 2020^7^ | £602.00 |
| ECG | 100.00 | 1.00 | £74.61 | NHS ref costs 2018/19^3^ | £74.61 |
| U&E test | 100.00 | 1.00 | £6.75 | NICE guidelines [NG45] ^8^ | £6.75 |
| **Total inpatient diagnosis cost (Patiromer)** | | | | | **£683.36** |
| **Total inpatient diagnosis cost (SoC)** | | | | | **£683.36** |
| **Outpatient diagnosis** | **0.00** |  | | | |
| **HK treatment** | **100.00** |  | | | |
| Inpatient day (Patiromer) | 100.00 | 3.0 | £602.00 | PSSRU 2020^7^ | £1,806.00 |
| Inpatient day (SoC) | 100.00 | 3.0 | £602.00 |  | £1,806.00 |
| Insulin | 100.00 | 2.00 | £0.60 | BNF NICE^14^; Actrapid assumed at 1 unit/kg per day | £1.20 |
| Glucose | 100.00 | 2.00 | £0.22 | PHS drug tariffs part 7 ^9^ | £0.44 |
| Calcium gluconate | 100.00 | 2.00 | £3.85 | NHS Generic Pharmaceuticals eMit^15^; 20mL A A H Pharmaceuticals Ltd | £7.70 |
| Salbutamol | 100.00 | 2.00 | £0.71 | PHS drug tariffs part 7 ^9^ | £1.42 |
| A & E and ambulance transport | 100.00 | 1.0 | £433.37 | NHS National Tariff Payment System 2018/19^3^ | £433.37 |
| **Total HK treatment cost (Patiromer)** | | | | | **£2,250.13** |
| **Total HK treatment cost (SoC)** | | | | | **£2,250.13** |
| **Total HK event cost (Patiromer)** | | | | | **£2,933.49** |
| **Total HK event cost (SoC)** | | | | | **£2,933.49** |
| ECG: electrocardiogram; HK: hyperkalaemia; U&E: urea and electrolytes.  The average daily dose of each temporising agent was obtained from Ahee et al.^16^ and Weisberg et al.^17^ | | | | | |

Table 17: Unit costs of temporising agents

| **Agent** | **Cost per pack** | **Total units per pack** | **Source** |
| --- | --- | --- | --- |
| **Insulin** | £7.48 | 1,000 IU | BNF NICE^14^; Actrapid assumed at 1 unit/kg per day |
| **Glucose** | £1.48 | 500 g bottle | PHS drug tariffs part 7 (1154711000001107)^9^ |
| **Calcium gluconate** | £19.26 | 100 mL, 10% solution for injection | NHS Generic Pharmaceuticals eMit^15^; 10mL A A H Pharmaceuticals Ltd |
| **Salbutamol** | £3.94-6.30 | 40 mg | PHS drug tariffs part 7 (3385611000001107, 3379211000001101)^9^ |
| eMit: Electronic Market Information; these values used as corresponding tariffs on the Public Health Scotland Part 7 list could not be identified.  *Currency code LE01A: haemodialysis for acute kidney injury, aged 19 years and over | | | |

Table 18: Dosing schedule and daily cost of temporising agents and temporary dialysis

| **Agent** | **Average daily dose** | **Cost per daily dose/use** | **Administration costs** | **Total cost per administration** |
| --- | --- | --- | --- | --- |
| **Insulin** | 80 IU | £0.60 | £0.00 | £0.60 |
| **Glucose** | 75 g | £0.22 | £0.00 | £0.22 |
| **Calcium gluconate** | 20 mL | £3.85 | £0.00 | £3.85 |
| **Salbutamol (nebulised)** | 10 mg | £0.71 | £0.00 | £0.71 |
| The average daily dose of each temporising agent and use of temporary haemodialysis was obtained from Ahee et al.^16^ and Weisberg et al.^17^ Salbutamol cost derived from nebulised form 10-20mg dose per NICE UK and Renal Association adult acute hyperkalaemia management guidelines.^18^ | | | | |

1. **Patiromer and SoC costs**

The cost of Patiromer treatment is based on an 8.4g daily dose costing £5.75 per day. SoC costs are assumed to be £0.00 and accounted for in the HK event costs.

1. **Inflation factors and inflated cost calculations**

Inflation factors were calculated using the Personal Social Services Research Unit (PSSRU) Hospital and community health services (HCHS) pay and price inflation index (Table 19).^19^ Inflation factors were used to inflate costs to 2019-20 values (Table 21).

Table 19: Inflation factors

| **Year** | **HCHS Index** | **Inflation factors** |
| --- | --- | --- |
| 2000-2001 | 196.5 | 1.61705 |
| 2001-2002 | 206.5 | 1.53874 |
| 2002-2003 | 213.7 | 1.48690 |
| 2003-2004 | 224.8 | 1.41348 |
| 2004-2005 | 232.3 | 1.36784 |
| 2005-2006 | 240.9 | 1.31901 |
| 2006-2007 | 249.8 | 1.27202 |
| 2007-2008 | 257.0 | 1.23638 |
| 2008-2009 | 267.0 | 1.19008 |
| 2009-2010 | 268.6 | 1.18299 |
| 2010-2011 | 276.7 | 1.14836 |
| 2011-2012 | 282.5 | 1.12478 |
| 2012-2013 | 287.3 | 1.10599 |
| 2013-2014 | 290.5 | 1.09380 |
| 2014-2015 | 293.1 | 1.08405 |
| 2015-2016 | 294.1 | 1.08027 |
| 2016-2017 | 300.4 | 1.05784 |
| 2017-2018 | 303.9 | 1.04571 |
| 2018-2019 | 310.9 | 1.02210 |
| 2019-2020 | 317.8 | 1.00000 |
| HCHS: Hospital and community health service  Inflation factors were calculated using the Personal Social Services Research Unit (PSSRU) Hospital and community health services (HCHS) pay and price inflation index.^19^ | | |

1. **Inflated cost calculations**

Table 21: Other inflated cost calculations

| **Input** | **Cost** | **Year** | **Source** | **Inflation factor** | **Cost**  **(2019/20)** |
| --- | --- | --- | --- | --- | --- |
| MACE | £4,400 (622) | 2010 | Kent et al.^5^ | 1.18299 | £5,205.14 |
| Hospitalisation HF | £2,308.00 | 2010-11 | Colquit et al.^6^ | 1.14836 | £2,650.41 |
| CKD 3 (annual) | £3,281.00 | 2011-12 | CG182^1^ | 1.12478 | £3,690.40 |
| CKD 4 (annual) | £3,281.00 | 2011-12 | CG182^1^ | 1.12478 | £3,690.40 |
| CKD 5 (pre-RRT) (annual) | £5,119.00 | 2011-12 | CG182^1^ | 1.12478 | £5,757.75 |
| Transplant  Maintenance (annual) | £4,851.00 | 2002-03 | CG125^4^ | 1.48690 | £7,212.94 |
| ECG | £72.00 | 2010/11 | NHS ref costs^3^ | 1.02210 | £74.61 |
| U&E | £6.00 | 2013/14 | NICE guidelines [NG45] ^8^ | 1.12478 | £6.05 |
| CKD: chronic kidney disease; FOI: freedom of information request; HF: heart failure; RRT: renal replacement therapy  Costs were inflated to 2019/20 values using the Personal Social Services Research Unit (PSSRU) Hospital and community health services (HCHS) pay and price inflation index.^19^  *NHS Blood and Transplant. Freedom of Information request response. Ref: FOI/VP/223037. 2012. | | | | | |

**References**

1. National Institute for Health and Care Excellence. Clinical guideline [CG182]: Chronic kidney disease in adults: assessment and management2015 08 December 2016. Available from: <https://www.nice.org.uk/guidance/cg182>.

2. Baboolal K, McEwan P, Sondhi S, Spiewanowski P, Wechowski J, Wilson K. The cost of renal dialysis in a UK setting—a multicentre study. Nephrology Dialysis Transplantation. 2008;23(6):1982-9.

3. Department of Health. NHS reference costs 2018 to 2019February 2021. Available from: <https://www.england.nhs.uk/national-cost-collection/>.

4. National Institute for Health and Care Excellence. Clinical guideline [CG125]: Chronic kidney disease (stage 5): peritoneal dialysis2011 08 December 2016. Available from: <https://www.nice.org.uk/guidance/cg125>.

5. Kent S, Briggs A, Eckermann S, Berry C. Are value of information methods ready for prime time? An application to alternative treatment strategies for NSTEMI patients. International journal of technology assessment in health care. 2013;29(04):435-42.

6. Colquitt JL, Mendes D, Clegg AJ, Harris P, Cooper K, Picot J, et al. Implantable cardioverter defibrillators for the treatment of arrhythmias and cardiac resynchronisation therapy for the treatment of heart failure: systematic review and economic evaluation. 2014.

7. Curtis L, Burns, A. Unit Costs of Health and Social Care 2016. University of Kent 2016 09 Feb 2017.

8. National Institute for Health and Care Excellence. NICE guideline [NG45]: Routine preoperative tests for elective surgery. Appendix M 2016 [

9. Public Health Scotland. Scottish drug tariffs: drugs and preparations with tariff prices (Part 7, March 2021) 2020 [Available from: <https://www.isdscotland.org/Health-Topics/Prescribing-and-Medicines/Scottish-Drug-Tariff/Drugs-and-Preparations-with-Tariff-Prices.asp>.

10. Vifor Pharma. OPAL-HK CSR. Data on file.; 2014.

11. Ponikowski P, Voors AA, Anker SD, Bueno H, Cleland JG, Coats AJ, et al. 2016 ESC Guidelines for the diagnosis and treatment of acute and chronic heart failure: The Task Force for the diagnosis and treatment of acute and chronic heart failure of the European Society of Cardiology (ESC) Developed with the special contribution of the Heart Failure Association (HFA) of the ESC. European heart journal. 2016;37(27):2129-200.

12. Haymarket Media Group Ltd. Monthly Index of Medical Specialities 2016 [Available from: <http://www.mims.co.uk/>.

13. National Institute for Health and Care Excellence. Technology appraisal guidance [TA599]: Sodium zirconium cyclosilicate for treating hyperkalaemia. 2019.

14. National Institute for Health and Care Excellence. British National Formulary 2020 [Available from: <https://bnf.nice.org.uk/>.

15. Department of Health. Drugs and pharmaceutical electronic market information (eMit) 2020 [Available from: <https://www.gov.uk/government/publications/drugs-and-pharmaceutical-electronic-market-information-emit>.

16. Ahee P, Crowe AV. The management of hyperkalaemia in the emergency department. Journal of accident & emergency medicine. 2000;17(3):188-91.

17. Weisberg LS. Management of severe hyperkalemia. Critical care medicine. 2008;36(12):3246-51.

18. Alfonzo A, Harrison A, Baines R, Chu A, Mann S, MacRury M. Clinical practice guidelines: Treatment of acute hyperkalaemia in adults 2020 [Available from: <https://renal.org/sites/renal.org/files/RENAL%20ASSOCIATION%20HYPERKALAEMIA%20GUIDELINE%202020.pdf>.

19. Curtis L. Personal Social Services Research Unit (PSSRU) Unit Costs of Health and Social Care 202008 December 2016. Available from: <https://www.pssru.ac.uk/project-pages/unit-costs/>.
