# Supplementary material for: A cost-effectiveness analysis of patiromer in the UK: evaluation of hyperkalaemia treatment and lifelong RAASi maintenance in chronic kidney disease patients with and without heart failure
Source: BMC Nephrol. 2023 Mar 9;24:47. doi: 10.1186/s12882-023-03088-3 (PMC9995261; doi:10.1186/s12882-023-03088-3)
Supplement: Supplementary file 3 — Additional file 3: This appendix provides details of utility and disutility input parameters utilised in the model. [file 12882_2023_3088_MOESM3_ESM.docx]

**Supplemental Appendix C**

This appendix provides details of utility and disutility input parameters utilised in the model.

1. **Overview**

Health-related utility values are utilised to characterise the quality of life of modelled patients and to estimate quality adjusted life-years (QALYs).

Health state utility values for CKD and NYHA health states are utilised by the model and are applied multiplicatively. For example, a patient in CKD stage 3 and NYHA stage II would have the health state utility of one multiplied by the other.

In contrast, transient events such as HK, hospitalisation and MACE incur disutilities in the cycle of incidence that are applied additively.

1. **Health state utility and impact of complications**

Health state utility values are applied multiplicatively and scaled to the current cycle length. Transient events are associated with a disutility that is applied additively in the cycle of incidence. Points of note in the application of utility values are as follows:

- If a patient has CKD and HF, both CKD and NYHA utility weights are applied.
- Disutilities associated with HK, hospitalisation and MACE events are applied in the cycle of incidence only (true for all cycles throughout the modelled horizon).
- Following the initiation of RRT, health state utility weights of dialysis and transplant are applied without adjustment for prior HF.

**Table 1** summarises health state utility weights applied according to CKD stage, dialysis, transplant, and NYHA classification and disutility associated with HK, MACE or hospitalisation events and dialysis complications.

Table 1: Health state utilities and disutilities of events

| **Parameter** | **Mean** | **SE** | **Source** |
| --- | --- | --- | --- |
| Health state utility: CKD 3 | 0.870 | 0.034 | Gorodetskaya et al.^1^ |
| Health state utility: CKD 4 | 0.850 | 0.029 | Gorodetskaya et al.^1^ |
| Health state utility: CKD 5 (pre RRT) | 0.570 | 0.057 | Lee et al.^2^ |
| Health state utility: Dialysis | 0.452 | 0.045^§^ | Lee et al.^2^* |
| Health state utility: Transplant | 0.710 | 0.019 | Lee et al.^2^ |
| Health state utility: NYHAI | 0.855 | 0.005 | Göhler et al.^3^ |
| Health state utility: NYHAII | 0.771 | 0.005 | Göhler et al.^3^ |
| Health state utility: NHYAIII | 0.673 | 0.006 | Göhler et al.^3^ |
| Health state utility: NYHAIV | 0.532 | 0.027 | Göhler et al.^3^ |
| Disutility: HK event | 0.000 | 0.000 | Section 3.4.4 |
| Disutility: MACE | -0.050 | 0.040 | Kent et al.^4^ |
| Disutility: Hospitalisation | -0.024 | 0.007 | Göhler et al.^3^ |
| Disutility: Dialysis complications | -0.060 | 0.006^§^ | NICE CG125^5^,  Sennfalt et al.^6^ |
| CKD: chronic kidney disease; HF: heart failure; HK: hyperkalaemia; MACE, Major adverse cardiac event; NYHA: New York Heart Association; SE: standard error  *Refer to Appendix F for information used to calculate the health state utility of dialysis  § SE values assumed 10% of mean | | | |

**References**

1. Gorodetskaya I, Zenios S, Mcculloch CE, Bostrom A, Hsu C-Y, Bindman AB, et al. Health-related quality of life and estimates of utility in chronic kidney disease. Kidney international. 2005;68(6):2801-8.

2. Lee AJ, Morgan CL, Conway P, Currie CJ. Characterisation and comparison of health-related quality of life for patients with renal failure. Current medical research and opinion. 2005;21(11):1777-83.

3. Göhler A, Geisler BP, Manne JM, Kosiborod M, Zhang Z, Weintraub WS, et al. Utility Estimates for Decision–Analytic Modeling in Chronic Heart Failure—Health States Based on New York Heart Association Classes and Number of Rehospitalizations. Value in Health. 2009;12(1):185-7.

4. Kent S, Briggs A, Eckermann S, Berry C. Are value of information methods ready for prime time? An application to alternative treatment strategies for NSTEMI patients. International journal of technology assessment in health care. 2013;29(04):435-42.

5. National Institute for Health and Care Excellence. Clinical guideline [CG125]: Chronic kidney disease (stage 5): peritoneal dialysis2011 08 December 2016. Available from: <https://www.nice.org.uk/guidance/cg125>.

6. Sennfalt K, Magnusson M, Carlsson P. Comparison of hemodialysis and peritoneal dialysis--a cost-utility analysis. Peritoneal Dialysis International. 2002;22(1):39-47.
