# Supplementary material for: A cost-effectiveness analysis of patiromer in the UK: evaluation of hyperkalaemia treatment and lifelong RAASi maintenance in chronic kidney disease patients with and without heart failure
Source: BMC Nephrol. 2023 Mar 9;24:47. doi: 10.1186/s12882-023-03088-3 (PMC9995261; doi:10.1186/s12882-023-03088-3)
Supplement: Supplementary file 4 — Additional file 4: This appendix provides details of additional results not presented in the main manuscript body. [file 12882_2023_3088_MOESM4_ESM.docx]

**Supplemental Appendix D**

This appendix provides details of additional results not presented in the main manuscript body.


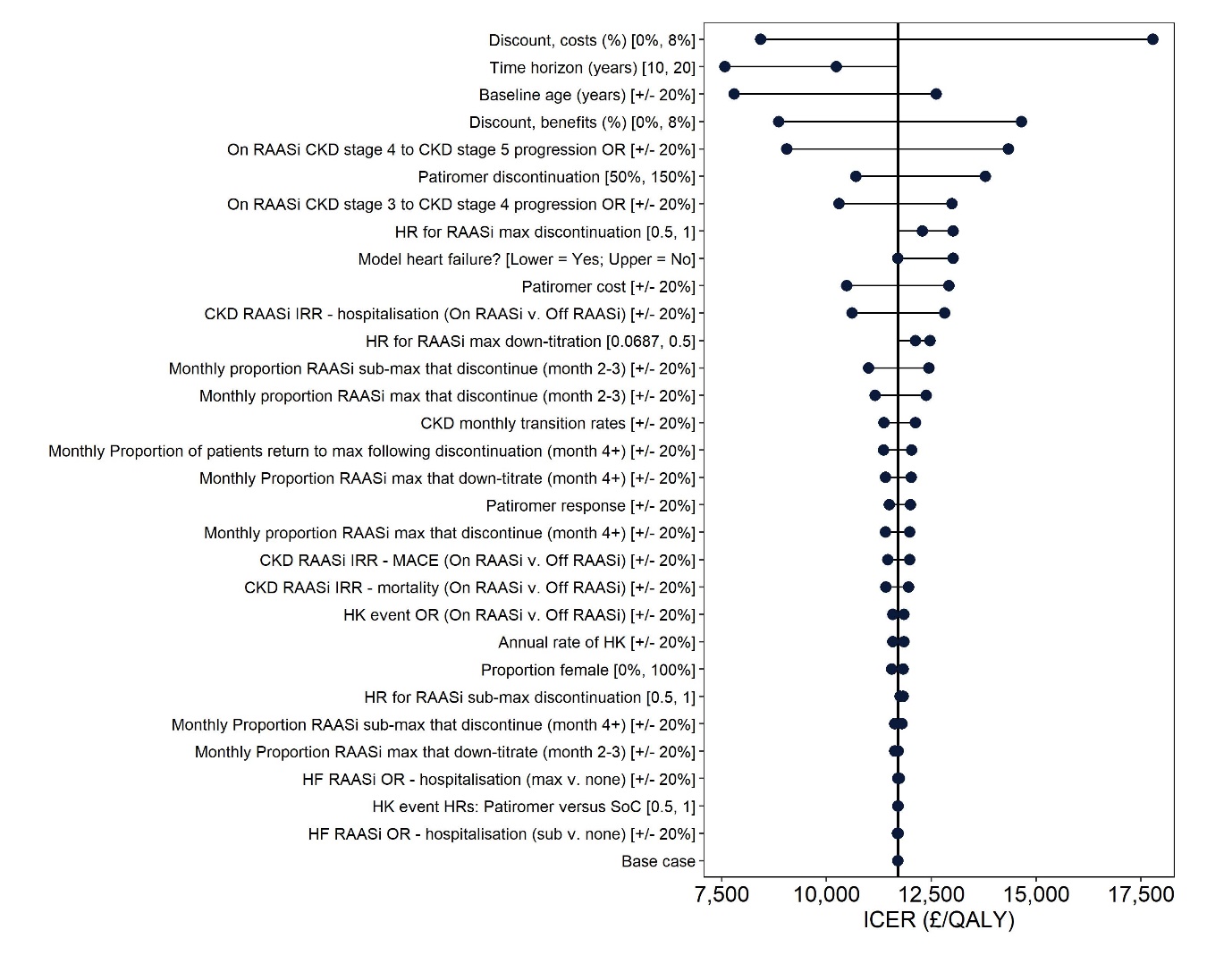


Figure 1: Deterministic sensitivity analysis
